# A feedback control mechanism governs the synthesis of lipid-linked precursors of the bacterial cell wall

**DOI:** 10.1101/2023.08.01.551478

**Published:** 2023-08-01

**Authors:** Lindsey S. Marmont, Anna K. Orta, Robin A. Corey, David Sychantha, Ana Fernández Galliano, Yancheng E. Li, Becca W.A. Baileeves, Neil G. Greene, Phillip J. Stansfeld, William M. Clemons, Thomas G. Bernhardt

**Affiliations:** 1 Department of Microbiology, Blavatnik Institute, Harvard Medical School, Boston, MA 02115.; 2 M.G. DeGroote Institute for Infectious Disease Research, David Braley Centre for Antibiotic Discovery, Department of Biochemistry and Biomedical Sciences, McMaster University, Hamilton, Canada.; 3 Division of Chemistry and Chemical Engineering, California Institute of Technology, Pasadena, California, USA; 4 School of Physiology, Pharmacology, and Neuroscience, University of Bristol, Bristol, UK; 5 School of Life Sciences and Department of Chemistry, University of Warwick, Warwick, UK; 6 Howard Hughes Medical Institute, Boston, United States

**Keywords:** Major: Biological Sciences, Minor: Microbiology, peptidoglycan, bacteriology, transferase, biochemistry

## Abstract

Many bacterial surface glycans such as the peptidoglycan (PG) cell wall, O-antigens, and capsules are built from monomeric units linked to a polyprenyl lipid carrier. How this limiting lipid carrier is effectively distributed among competing pathways has remained unclear for some time. Here, we describe the isolation and characterization of hyperactive variants of *Pseudomonas aeruginosa* MraY, the essential and conserved enzyme catalyzing the formation of the first lipid-linked PG precursor called lipid I. These variants result in the elevated production of the final PG precursor lipid II in cells and are hyperactive in a purified system. Amino acid substitutions within the activated MraY variants unexpectedly map to a cavity on the extracellular side of the dimer interface, far from the active site. Our structural evidence and molecular dynamics simulations suggest that the cavity is a binding site for lipid II molecules that have been transported to the outer leaflet of the membrane. Overall, our results support a model in which excess externalized lipid II allosterically inhibits MraY, providing a feedback mechanism to prevent the sequestration of lipid carrier in the PG biogenesis pathway. MraY belongs to the broadly distributed polyprenyl-phosphate N-acetylhexosamine 1-phosphate transferase (PNPT) superfamily of enzymes. We therefore propose that similar feedback mechanisms may be widely employed to coordinate precursor supply with demand by polymerases, thereby optimizing the partitioning of lipid carriers between competing glycan biogenesis pathways.

Bacterial cells surround themselves with a complex envelope that is essential for their integrity and shape. The envelopes of gram-negative (diderm) bacteria also serve as a formidable barrier against the entry of drug molecules, providing organisms like *Escherichia coli* and *Pseudomonas aeruginosa* with a relatively high intrinsic resistance to antibiotics^1,2^. Understanding how these bacteria construct their envelope and regulate the assembly process therefore promises to aid in the identification of new vulnerabilities in surface biogenesis to target for antibiotic development.

The diderm envelope consists of two membranes: a cytoplasmic (inner) membrane and an asymmetric outer membrane (OM) with an inner leaflet of phospholipids and an outer leaflet composed of lipopolysaccharide (LPS)^2^. The LPS molecule consists of a Lipid A moiety, a core oligosaccharide, and a long polysaccharide chain called the O-antigen (O-Ag) that varies in composition between different strains and species^3^. Between the inner and outer membranes is the periplasmic space where the peptidoglycan (PG) cell wall is assembled. The PG layer is the essential stress bearing portion of the envelope that protects cells from osmotic lysis. It is constructed from glycan strands with repeating *N*-acetylglucosamine (GlcNAc) and *N*-acetylmuramic acid (MurNAc) sugars that are crosslinked by peptide stems attached to MurNAc forming the interconnected meshwork that encases the inner membrane^4^.

Surface glycans like PG and O-Ag are polymerized from monomeric building blocks attached to polyprenyl lipids via a pyrophosphate linkage. The lipid carrier is regenerated during polymerization, making it available for the continued production of monomer units to support synthesis of growing polymers^5,6^. This synthetic strategy is conserved throughout biology with uses ranging from surface glycan biogenesis in microbes to the production of N-linked glycans in eukaryotic cells^7–10^. In any given organism, a common polyprenyl lipid carrier is used to produce multiple different glycans^7^. The concentration of these carriers is limiting^8^, suggesting that their utilization to produce monomer units for different pathways must be coordinated with the corresponding glycan polymerization process. Otherwise, excess accumulation of monomer units for one polymer will sequester the limiting carrier, indirectly inhibiting the production of other glycans that require the carrier for their biogenesis. Such precursor sequestration can have significant detrimental consequences for the cell envelope^8–11^. Despite the importance of efficient carrier utilization for the balanced synthesis of different surface glycans, the underlying mechanism has remained elusive.

The membrane-anchored precursor for PG biosynthesis is lipid II. Its synthesis begins in the cytoplasm, where multiple enzymes (MurA-F) sequentially assemble the activated sugar uridine diphosphate-MurNAc-pentapeptide (simplified as UM5)^4^. The phospho-MurNAc-pentapeptide moiety from this intermediate is then transferred to the lipid carrier undecaprenyl phosphate (C55P) at the inner face of the cytoplasmic membrane by the essential integral membrane enzyme MraY, generating the penultimate PG precursor, lipid I. The peripheral membrane enzyme MurG then transfers GlcNAc from UDP-GlcNAc to lipid I, forming the lipid II molecule, which contains the basic monomeric unit of PG linked to the C55 lipid by a pyrophosphate. Following its synthesis, lipid II is transported across the cytoplasmic membrane by the flippase MurJ where it can then be polymerized and crosslinked by PG synthases to form the cell wall matrix^12^.

There are two general types of PG synthases, and bacterial cells typically encode multiple members of each. The class A penicillin-binding proteins (aPBPs) are one type. They are single-pass membrane proteins with a large extracytoplasmic domain that possesses both PG glycosyltransferase (PGTase) activity to polymerize lipid II and transpeptidase (TPase) activity to crosslink the nascent glycans into the mature wall^13,14^. The second type of synthase is formed by a complex between a SEDS (shape, elongation, division, and sporulation) family PGTase and a class B PBP (bPBP) with TPase activity. The SEDS-bPBP complexes form the essential PG synthases of the cell elongation and division machineries whereas the aPBPs are thought to fortify a foundational PG structure laid down by the morphogenic SEDS-bPBP systems^15–17^.

This investigation started with the study of *P. aeruginosa* mutants with a conditionally lethal defect in aPBP activity^18^. We isolated suppressors encoding an altered MraY enzyme with a T23P substitution [MraY(T23P)] that restored the growth of these cells in the non-permissive condition. Our characterization of this and other related MraY variants supports a model in which MraY is feedback inhibited by the accumulation of flipped lipid II, limiting the synthesis of PG precursors when their supply exceeds the synthetic capacity of PG synthases.

## RESULTS

### An MraY variant rescues a lethal aPBP synthase defect

**Figure 1.**
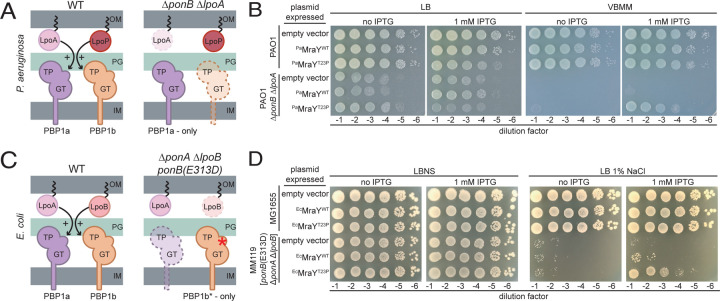
MraY(T23P) restores growth to strains defective in PG biosynthesis. Schematic representation of the aPBPs and their outer membrane lipoprotein activators in *Pseudomonas aeruginosa* (**A**) and *Escherichia coli* (**C**). Ten-fold serial dilutions of cells of the indicated *P. aeruginosa* (**B**) or *E. coli* (**D**) strains harboring expression plasmids producing the indicated MraY variant. Dilutions were plated on the indicated medium with or without IPTG to induce the production of MraY variants as indicated. Abbreviations: OM, outer membrane; PG, peptidoglycan; IM, inner membrane; GT, glycosyltransferase; TP, transpeptidase; LB, lysogeny broth; LBNS, LB with no added NaCl; VBMM, Vogel-Bonner minimal medium; IPTG, isopropyl-B-D-1-thiogalactopyranoside.

*P. aeruginosa* produces two aPBPs, ^*Pa*^PBP1a and ^*Pa*^PBP1b, encoded by the *ponA* and *ponB* genes, respectively. These PG synthases require cognate OM lipoprotein activators to function properly^18,19^. PBP1a is activated by ^*Pa*^LpoA and ^*Pa*^PBP1b is activated by ^*Pa*^LpoP^18^ (Fig. 1A). A Δ*ponB* Δ*lpoA* mutant relies on an unactivated PBP1a enzyme for growth (Fig. 1A). We therefore refer to the strain as a PBP1a-only mutant for simplicity. Such mutants are viable on rich medium (lysogeny broth, LB), but were found to have severe growth defects on a defined minimal medium (Vogel-Bonner minimal medium, VBMM)^18^. Spontaneous suppressors supporting the growth of the PBP1a-only mutant on VBMM medium were isolated to uncover new insights into PG synthesis regulation. Several of these mutants were found to encode variants of ^*Pa*^PBP1a, and we previously reported that they bypass the ^*Pa*^LpoA requirement for ^*Pa*^PBP1a function by activating the PG synthase^20^. Thus, the growth defect of the PBP1a-only strain on VBMM is caused by a deficit of aPBP activity. Here, we report the identification of a new class of suppressor with a mutation in ^*Pa*^*mraY* encoding an enzyme variant with a T23P substitution [^*Pa*^MraY(T23P)] that alleviates the growth defect of the Δ*ponB* Δ*lpoA* strain.

To confirm the suppression of the PBP1a-only growth defect by the MraY variant, ^*Pa*^*mraY*(WT) or ^*Pa*^*mraY*(T23P) were expressed from a multicopy plasmid under the control of an IPTG-inducible promoter in a wild-type *P. aeruginosa* (strain PAO1) or a Δ*ponB* Δ*lpoA* background (Fig. 1B). Overexpression of ^*Pa*^*mraY*(WT) in the wild-type PAO1 strain did not appreciably affect growth on either LB or VBMM nor did it rescue the lethal phenotype of the Δ*ponB* Δ*lpoA* mutant on VBMM (Fig. 1B). Consistent with the results of the genetic selection, expression of ^*Pa*^*mraY*(T23P) restored growth of the PBP1a-only mutant on VBMM with as little as 25 µM of inducer promoting significant growth on these non-permissive conditions (Fig. 1B and Fig. S1). Notably, in addition to rescuing growth of the mutant on VBMM, overexpression of ^*Pa*^*mraY*(T23P) caused a mild growth defect in both PAO1 and Δ*ponB* Δ*lpoA* backgrounds when cells were grown on rich medium (Fig. 1B and Fig. S1) (see below). Substitution of the catalytic residue D267 with Ala in the active site of ^*Pa*^MraY(T23P) eliminated the toxicity of the variant when it was overproduced in PAO1 cells on LB and greatly reduced the suppression activity in Δ*ponB* Δ*lpoA* cells on VBMM (Fig. S1). Furthermore, VSVG-tagged derivatives of ^*Pa*^MraY(WT) and ^*Pa*^MraY(T23P) were found to accumulate to similar levels in cells by immunoblot analysis with the tagged ^*Pa*^MraY(T23P) variant promoting better growth of Δ*ponB* Δ*lpoA* cells on VBMM than the tagged wild-type protein (Fig. S2). Thus, the suppression activity of the ^*Pa*^MraY(T23P) variant is not due to increased accumulation of the enzyme. Rather, the results suggest that the T23P change alters MraY activity to promote the growth of the aPBP deficient strain on VBMM and impair growth of both mutant and wild-type strains on LB when it is overexpressed.

*E. coli* also encodes aPBPs, ^*Ec*^PBP1a and ^*Ec*^PBP1b, controlled by OM lipoprotein activators ^*Ec*^LpoA and ^*Ec*^LpoB, respectively (Fig. 1C)^19,21^. We previously described an *E. coli* strain lacking ^*Ec*^PBP1a and ^*Ec*^LpoB that relies on a LpoB-bypass variant of ^*Ec*^PBP1b [^*Ec*^PBP1b(E313D)] as its only aPBP (Fig. 1C)^22^. Like the *P. aeruginosa* Δ*ponB* Δ*lpoA* strain, this *E. coli* mutant has a conditional growth defect caused by a deficit in aPBP activity. It grows on LB without added NaCl (LBNS) but is inviable on LB with 1% NaCl. Overproduction of *E. coli* MraY(T23P) [^*Ec*^MraY(T23P)] but not wild-type ^*Ec*^MraY suppressed the growth defect of this aPBP-deficient *E. coli* strain on LB 1% NaCl (Fig. 1D). Therefore, an MraY(T23P) variant suppresses an aPBP defect in two distantly related gram-negative bacteria, suggesting that its properties are conserved.

### MraY(T23P) is activated and increases lipid II accumulation in cells

**Figure 2.**
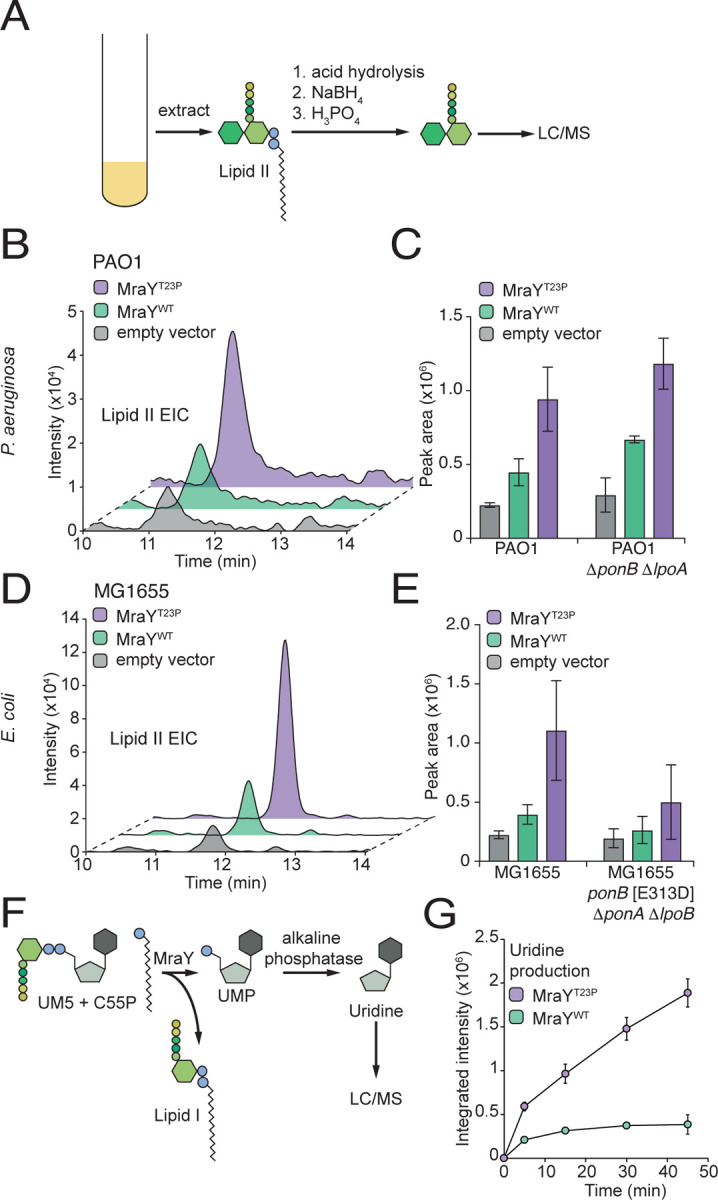
Cells expressing MraY(T23P) accumulate lipid II. (**A**) Schematic representation of the method used to isolate and analyze lipid II from bacterial cells. (**B-D**) Representative extracted ion chromatograms of lipid II (EIC) and quantification of EICs for *P. aeruginosa* (**B,C**) or *E. coli* (**D,E**) strains expressing the indicated MraY variant. Three independent replicates of the extractions were performed and lipid II levels quantified using the area of the peak from the extracted ion chromatogram using the Agilent software. Error bars represent the standard deviation. For MraY(T23P) vs MraY(WT) in PAO1 P<0.05 PAO1 Δ*ponB* Δ*lpoA* P<0.01, in PAO1, MG1655 P<0.05, MG1655 Δ*ponA* Δ*lpoB ponB*[E313D], not significant. (**F**). Schematic representation of the MraY enzyme assay. (**G**) Representative time course showing the production of uridine in assays containing purified MraY or MraY(T23P) as indicated. The assay was repeated at least twice with two independent preparations of protein. Error bars represent standard deviation of reactions performed in duplicate. Abbreviations: NaBH_4_, sodium borohydride; H_3_PO_4_, phosphoric acid; LC/MS, liquid chromatography mass spectrometry; UM5, UDP-MurNAc-pentapeptide; C55P, undecaprenylphosphate.

MraY uses UM5 and C55P to form the first lipid-linked PG precursor lipid I, which is then converted to the final precursor lipid II by MurG. We reasoned that MraY(T23P) might overcome the aPBP-deficiency in mutants of *P. aeruginosa* and *E. coli* by increasing the concentration of the synthase substrate lipid II to compensate for the poorly activated aPBP in these cells. To investigate this possibility, we measured the concentration of lipid II in *P. aeruginosa* and *E. coli* cells overproducing MraY(WT) or MraY(T23P). Exponentially growing cultures were normalized by optical density, and the cells were harvested and extracted for lipid-linked PG precursors (Fig. 2A). The extract was subjected to acid hydrolysis to release the disaccharide-pentapeptide from undecaprenyl-pyrophosphate (C55PP), and the soluble disaccharide-pentapeptide was subsequently detected by liquid chromatography/mass spectrometry (LCMS) as a measure of lipid II concentration (Fig. 2B-E). In both the wild-type and aPBP deficient mutant backgrounds, MraY(WT) overproduction led to an approximately twofold increase in lipid II levels relative to an empty vector control (Fig. 2C and 2E). The increase was another twofold higher for cells overproducing MraY(T23P) (Fig. 2C and 2E). We observed similar trends monitoring lipid I levels, but the increase in lipid I levels in cells producing MraY(T23P) relative to MraY(WT) was not nearly as pronounced compared to the change in lipid II levels (Fig. S3). These results suggest that the altered MraY enzyme is more active than wild-type and that the ability to promote the accumulation of higher lipid II levels indeed underlies the suppression of aPBP defects.

To assess the effect of the T23P substitution on MraY activity directly, FLAG-tagged derivatives of ^*Pa*^MraY(WT) and ^*Pa*^MraY(T23P) were heterologously expressed in *E. coli* and affinity purified for biochemical assays. The reaction was followed by monitoring the production of uridine derived from alkaline phosphatase treatment of the UMP product (Fig. 2F). Using this assay, the ^*Pa*^MraY(T23P) variant was found to be significantly more active than ^*Pa*^MraY(WT). At the conclusion of the time-course, approximately five times more uridine was detected in reactions containing ^*Pa*^MraY(T23P) than those with ^*Pa*^MraY(WT) (Fig. 2G). We conclude that the T23P substitution generates a hyperactive MraY, leading to elevated lipid II production in cells.

### Misregulated MraY disrupts O-antigen synthesis

The results thus far suggest that ^*Pa*^MraY(T23P) makes more lipid-linked PG precursors than normal, leading to their hyperaccumulation. In the PBP1a-only *P. aeruginosa* strain, this increase in substrate supply suppresses the lethal aPBP deficiency. We wondered whether excess lipid II production and the resulting sequestration of C55P in this building block might also indirectly impede the synthesis of other surface glycans built on the lipid carrier like O-Ag. A clue that this was the case came from the growth defect on LB medium of the wild-type PAO1 strain caused by ^*Pa*^MraY(T23P) but not ^*Pa*^MraY(WT) overproduction (Fig. 1B and Fig. S1). Notably, this strain produces R2-pyocin, a lethal phage tail-like bacteriocin that uses a receptor located within the LPS core to engage target cells^23,24^. *P. aeruginosa* is resistant to killing by its own R2-pyocin because it decorates its LPS with O-Ag that masks the R2-pyocin receptor. Defects in the O-Ag synthesis pathway therefore result in susceptibility to R2-pyocin self-killing^25^. The connection between O-Ag and R2-pyocin activity suggested to us that the growth phenotype induced by ^*Pa*^MraY(T23P) overproduction on LB medium may be caused by a decrease in O-Ag production and increased R2-pyocin self-intoxication. To test this possibility, we examined the effect of ^*Pa*^MraY(T23P) overproduction in a strain deleted for the R2-pyocin gene cluster (*PA0615-PA0628*). Strikingly, unlike wild-type cells, the mutant incapable of making R2-pyocin was largely unaffected by the overproduction of ^*Pa*^MraY(T23P) (Fig. S4A), indicating that the growth defect caused by the altered enzyme was largely due to R2-pyocin killing. This result suggested that O-Ag synthesis is reduced when lipid II synthesis is hyperactivated in cells producing ^*Pa*^MraY(T23P). Analysis of the LPS produced by these cells confirmed that they indeed have reduced levels of O-Ag. They made approximately 30% less O-Ag compared to cells expressing ^*Pa*^MraY(WT) (Fig. S4B-C). These results suggest that ^*Pa*^MraY(T23P) may be insensitive to a regulatory mechanism limiting the steady-state accumulation of lipid-linked PG precursors to prevent the impairment of competing pathways utilizing the C55P carrier.

### The extracytoplasmic side of the MraY dimer interface may be a regulatory site

**Figure 3.**
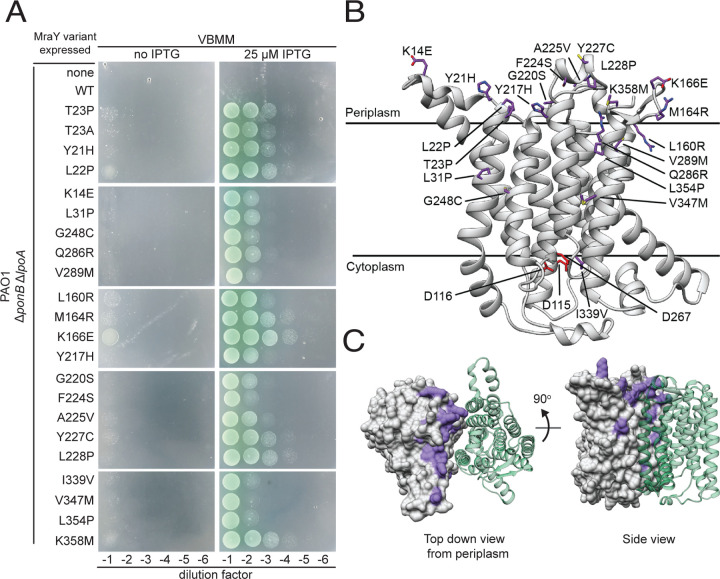
Amino acid substitutions in hyperactive MraY variants localize to the extracytoplasmic surface of the dimer interface. (**A**) Ten-fold serial dilutions of *P. aeruginosa* Δ*ponB* Δ*lpoA* cells harboring expression plasmids producing the indicated MraY variant were plated on VBMM with or without IPTG to induce the MraY variants as indicated. (**B**) Structural model of *P. aeruginosa* MraY created using AlphaFold^28^ in cartoon viewed from the plane of the membrane. Residues altered in hyperactive variants tested in (**A**) are shown in stick representation (purple), while those residues previously implicated in catalysis are shown in red. (**C**) Structural model of the MraY dimer created using AlphaFold^28^. Surface representation of one protomer is shown in grey with the residues altered in hyperactive variants colored in purple. The other protomer is shown in cartoon representation (green) for simplicity. Left, the periplasmic view of the dimer; Right, view from the plane of the membrane.

MraY is a polytopic membrane protein with ten transmembrane helices and an N-out, C-out topology^26^. The structure of the enzyme from *Aquifex aeolicus* revealed that it forms a dimer with most of the monomer-monomer contacts made between the N- and C-terminal helices^26^. Notably, the T23 residue lies near the dimer interface on the extracytoplasmic side of MraY. We therefore wondered whether other substitutions in this area might also activate the enzyme. To test this possibility, a mutagenized copy of ^*Pa*^*mraY* under the control of an IPTG inducible promoter was transformed into the Δ*ponB* Δ*lpoA P. aeruginosa* strain. The resulting transformants were then selected on VBMM in the presence of IPTG to identify MraY variants that rescue the aPBP deficiency. Twenty-one suppressing clones were isolated that each contained a single point mutation in the plasmid-borne copy of *mraY* (Fig. 3A). The positions of these substitutions were mapped onto a model of the ^*Pa*^MraY structure generated using AlphaFold^27,28^. Strikingly, all changes were located proximal to the dimer interface, with a majority positioned on the extracytoplasmic side of the protein far from the active site, which is located on the cytoplasmic side of the enzyme (Fig. 3B-C, Table S1). Overall, our genetic and biochemical results implicate the extracytoplasmic region of MraY near the dimer interface as a potential regulatory site for the enzyme.

### A potential binding site for flipped lipid II within the MraY dimer interface

Both the *A. aeolicus* and *Enterocloster boltae* MraY crystal structures revealed the presence of a cavity located at the dimer interface that is lined by hydrophobic residues^26,29^. The authors concluded that electron density within this tunnel could accommodate a cylindrical molecule that is too long to be detergent from the sample preparation^26^. Instead, they suggested that this electron density could accommodate one or more lipid molecules. Although it has been speculated to be C55P^26^, the identity of the lipid has remained unclear. Additionally, a recent study identified lipid molecules co-purifying with MraY using native mass-spectrometry^30^. The most abundant species were the C55P substrate and lipid I product, but peaks corresponding to C55PP, cardiolipin, and lipid II were also detected^30^. Thus, MraY likely binds a lipid molecule within the dimer interface near residues we have implicated in controlling the activity of the enzyme.

**Figure 4.**
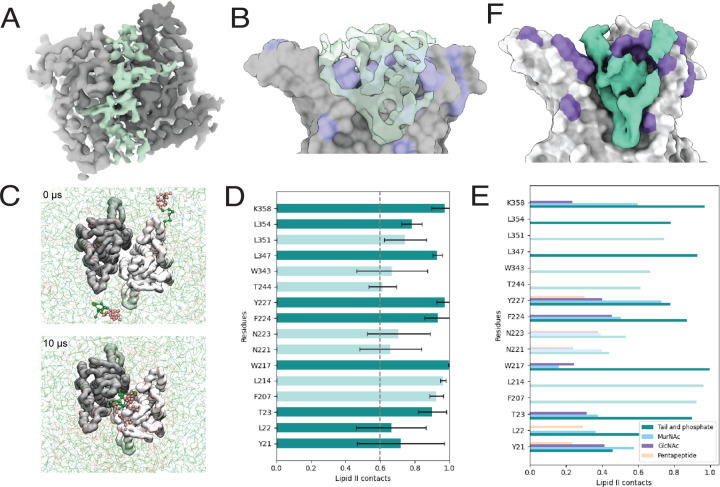
Identification of a potential lipid II binding site in MraY. (**A**) Periplasmic view of the cryo-EM structure of the ^*Ec*^MraY(T23P) dimer within the YES complex shown in surface representation, with the unmodeled electron density shown (green). (**B**) As in *A*, membrane view of the electron density within the dimer interface of the ^*Ec*^MraY dimer with the foreground MraY removed. (**C**) Top view of the MraY dimer in a mixed CG membrane. Two lipid II molecules (highlighted as green, gold and pink spheres) freely enter the MraY cavity during unbiased MD simulations. In 8/9 repeats, 2 or 3 lipid I or II molecules bind the cavity. In the last repeat, one lipid II and one C55P molecule bind. (**D**) Lipid II contacts with MraY residues that interact with lipid II for over 60% of atomistic MD simulations. Error bars represent standard error from 5 repeats. Darker green bars represent residues altered in hyperactive variants. (**E**) Lipid II contacts with MraY residues by part of lipid II that is interacting (tail & phosphate, MurNAc, GlcNAc or pentapeptide). Residues shown are same as those in Fig 4D. Darker bars represent residues altered in hyperactive variants. (**F**) Average density of lipid II molecules (green) from atomistic MD simulations of MraY (grey) bound to lipid II. Shown as inside view of dimer interface, where only one monomer of MraY is shown and residues altered in hyperactive variants are colored in purple.

Clues to the potential identity of the lipid bound at the MraY dimer interface came from structural analysis of ^*Ec*^MraY in complex with a phage-encoded inhibitor (protein E) and the *E. coli* chaperone SlyD (the YES complex)^31^. The cryo-EM structure of the YES complex containing wild-type ^*Ec*^MraY was recently reported^31^, and these methodologies were used to obtain the structure of ^*Ec*^MraY(T23P) within the same complex (Fig. S5, Table S2). In both cases, electron density was observed at the MraY dimer interface. Focused refinement of MraY alone in the ^*Ec*^MraY(T23P) complex significantly improved the potential lipid density at the MraY dimer interface (Fig. 4A-B). As in previous *A. aeolicus* and *E. boltae* MraY structures, this electron density fills the hydrophobic cavity found at the MraY dimer interface. However, we uniquely observed this electron density extending into the periplasmic space above the MraY molecules where the environment is more hydrophilic (Fig. 4A-B). Although structural refinement alone could not conclusively identify the lipid within the dimer, the size of the electron density extending into the periplasmic space is consistent with a large head-group such as the disaccharide-pentapeptide found on lipid II.

To assess whether a lipid II molecule could enter the hydrophobic cavity of the MraY dimer, we used molecular dynamics (MD) simulations. In the first set of simulations, we used the structure of the *E. coli* MraY dimer from the YES complex (PDBID 8G01)^31^ embedded in a lipid bilayer [palmitoyl-2-oleoyl-*sn-*glycero-3-phosphoethanolamine (POPE), palmitoyl-2-oleoyl-*sn*-glycero-3-phosphoglycerol (POPG) and cardiolipin (CDL)] containing C55P, C55PP, lipid I, or lipid II with hydrophilic head-groups oriented towards what would be the periplasmic side of the membrane. Using course-grained MD simulations we observed that in almost all runs, lipid I and lipid II molecules spontaneously entered the central cavity, where typically two molecules would occupy the cavity (Fig. 4C, Movie S1). POPE and POPG never entered the cavity, while C55P and C55PP would occasionally enter the cavity at approximately 4% and 10% of the simulation time (Movie S2). Lipid I and Lipid II would remain stably bound for at least several µs (Movie S1), reflected by the K_off_ values for lipid I and lipid II of 0.218 µs^-1^ and 0.206 µs^-1^, respectively. In similar experiments where lipid I and lipid II are omitted, a single CDL molecule or C55P molecule will enter the cavity (Movie S2), although in the majority of simulations, no lipid enters the cavity. These were much shorter lasting interactions, with K_off_ values of 3.976 µs^-1^ for C55P and 1.297 µs^-1^ for CDL. Although lipid I and lipid II have similar K_off_ values in these simulations, lipid I is not found in the periplasmic leaflet of the inner membrane. Therefore the simulations with lipid I are not likely to reflect a physiologically relevant binding event. Instead, lipid II is the best candidate for the native ligand due to its strong and long-lasting interaction. Notably, the bound lipid II molecules in the simulations make extensive contacts with the MraY dimer, with many residues contacting the bound lipids for 100% of the MD simulations (Fig 4D, Fig. S6). These residues include several that were identified in the mutational analysis as being hyperactive (Fig. 3A-B). To investigate the interaction in more detail, a pose of the *E. coli* MraY dimer with two bound lipid II molecules was converted to an atomistic description for further MD analysis. The data show that the lipid II molecules are stable in the central cavity with the isoprenyl chains adopting a curved orientation. The result predicts contacts between the MurNAc sugar and MraY that include several residues where substitutions were identified in our screen (Y21, L22, T23, W217, F224, Y227, and K358) (Fig. 4E-F). Together, these data indicate that C55P-linked lipids can spontaneously enter a previously empty MraY dimer interface cavity and that externalized lipid II is likely to be the ligand bound in the potential regulatory site identified in the genetic and biochemical analyses.

## DISCUSSION

Bacterial surfaces contain multiple types of glycan and other polymers that are required for cellular integrity and/or barrier function. Although most of the proteins involved in the synthesis of major surface components are known, how the biogenesis of these molecules is regulated to efficiently distribute shared precursors like the C55P lipid carrier among competing synthesis pathways remains poorly understood. In this report, we uncover a mechanism governing the activity of MraY, the essential enzyme catalyzing the first membrane step in the PG synthesis pathway in which C55P is consumed to form lipid-linked PG precursors. This regulation is likely to play an important role in the efficient distribution of C55P among glycan biogenesis pathways that utilize the limiting carrier.

**Figure 5.**
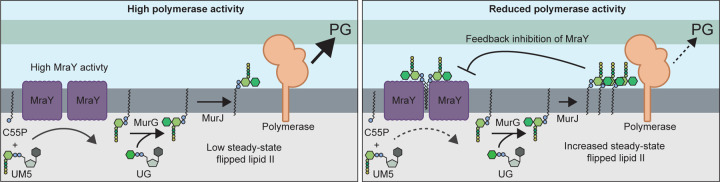
Model for feedback regulation of MraY by flipped lipid II. Shown are schematics summarizing the model for MraY regulation. Left: When PG polymerase activity is high, flipped lipid II is consumed at a rate proportional to its production such that steady-state levels of the precursor remains low and MraY activity is unimpeded. Right: When PG polymerase activity is reduced due to changes in growth conditions or other perturbations, lipid II will be produced faster than it is consumed, resulting in the accumulation of elevated levels of flipped lipid II. Higher levels of the precursor promote its binding to MraY dimers, reducing their activity in order to bring lipid II supply back in balance with demand by the polymerases. See text for details. Abbreviations: C55P, undecaprenylphosphate; UM5, UDP-MurNAc-pentapeptide; UG, UDP-GlcNAc; PG, peptidoglycan.

The first clue that MraY is regulated came from the discovery that an *mraY(T23P)* mutant can suppress an aPBP deficiency in both *P. aeruginosa* and *E. coli*. The aPBP deficient strains encode a single aPBP lacking its required activator. Prior work with these strains suggests that their conditionally lethal growth phenotypes are caused by poor PG synthesis efficiency resulting from the synthase having a reduced affinity for lipid II in the absence of its activator^20^. Accordingly, we infer that MraY(T23P) suppresses this problem by raising the steady state level of lipid II to overcome the substrate binding limitations of the unactivated aPBP. The ability of the altered MraY to increase lipid II levels indicates a role for the enzyme in regulating the maximum level of lipid II in cells. We propose that this control is mediated via feedback inhibition of MraY by externalized lipid II (Fig. 5).

In support of the feedback inhibition model, the biochemical results with purified enzymes indicate that the observed regulation is intrinsic to MraY and does not require additional proteins. The MraY(T23P) variant, which is apparently less sensitive to regulatory control, showed much greater activity *in vitro* than the wild-type enzyme. At first glance, this result may seem incompatible with the proposed feedback control given that the product of the reaction is lipid I with its head-group in the cytoplasm, not externalized lipid II. However, because the reactions are performed in detergent, the lipid I formed in the reaction is likely capable of reorienting in the micelles to mimic a periplasmic orientation. Although externalized lipid I is not observed *in vivo*, the MD simulations predict that both flipped lipid I and lipid II are capable of binding at the MraY dimer interface. It is therefore reasonable to interpret the biochemical results in the context of a feedback inhibition model with MraY(WT) activity leveling off early in the time course due to feedback control. By contrast, we infer that MraY(T23P), with its substitution in the proposed binding site for flipped lipid II, is insensitive to feedback control and therefore displays robust activity in the assay. Another factor that is likely to contribute to the biochemical results is the co-purification of lipid II with the purified enzymes, which according to the model would be expected to further reduce the activity of MraY(WT) relative to MraY(T23P). Importantly, the activity for the wild-type enzyme was already so low that it was not possible to directly test for feedback inhibition via the addition of purified lipid II to the enzyme. Nevertheless, based on the logic above and the totality of the results presented, feedback inhibition of MraY by flipped lipid II provides one of the simplest and most straightforward explanations for our findings.

Although additional experiments are required to further investigate the possible feedback regulation of MraY, it is a compelling model because it suggests a mechanism by which cells can balance the supply of flipped lipid II precursor with the activity of the PG synthases that use it to build the cell wall (Fig. 5). We propose that when PG synthases are highly active, the steady state level of lipid II remains low such that MraY is functioning near its maximum activity to continue supplying lipid-linked PG precursors (Fig. 5, left panel). However, when the supply of lipid II exceeds the capacity of the PG synthases to use it, either transiently or due to a change in growth conditions, the steady state level of lipid II will rise such that it begins binding MraY dimers to inhibit their activity and reduce flux through the lipid stages of PG precursor production until supply more closely matches demand (Fig. 5, right panel). Such feedback control would prevent excess C55P from being sequestered in PG precursors when they are not needed, making more of the lipid carrier available to other glycan synthesis pathways for their efficient operation. Accordingly, *P. aeruginosa* cells with an activated MraY variant, which is presumably less sensitive to feedback control, display reduced ability to make O-Ag, rendering them susceptible to self-intoxication by their encoded pyocins (Fig. S4B, C).

The location of the amino acid substitutions in MraY that suppress aPBP defects combined with the structural and MD analysis suggest a mechanism by which the enzyme may be regulated by lipid II binding. Many of the MraY substitutions that overcome the PG synthesis defects of the PBP1a only strain localize to the extracytoplasmic surface of the protein distal to, and on the other side of the membrane from, the active site. These changes flank the opening of a deep hydrophobic pocket at the MraY dimer interface. In the cryo-EM structure of MraY within the YES complex^31^, we observe an MraY dimer with electron density at this interface as observed in prior X-ray crystal structures^26,29^. However, in our structure, this density not only fills the pocket but also extends into the extracytoplasmic opening. This density in the extracytoplasmic space is large enough to correspond to a head-group of flipped lipid II. Accordingly, MD simulations indicate the capacity of MraY dimers to bind two molecules of flipped lipid II with contacts between the protein and the MurNAc sugar that likely provide specificity for externalized lipid II binding over C55PP or C55P. Notably, the head-groups of the lipid II binding substrates remain relatively flexible in the simulations (Movie S1, Fig. S7), which likely accounts for our inability to further refine the structure of the bound molecules by cryo-EM.

The MD simulations predict conformational changes in the MraY dimers associated with lipid II binding that increase the distance between the 6^th^ transmembrane helix (TM6) of each monomer in the dimeric structure and alter the position of the 9^th^ transmembrane helix (TM9) (Fig. S8A-D). Similarly, the distance between a periplasmic helix (residues 221–228) from each monomer is also increased (Fig. S8C-F). These changes are reminiscent of the conformational difference between MraY in the YES complex relative to the free MraY structure from *A. aeolicus*^26^. When the structures are aligned on one monomer, the second monomer in the YES complex^31^ is tilted relative to its partner in the *A. aeolicus* dimer^26^ resulting in the opening the periplasmic cavity and tightening the interface at the cytoplasmic side of the enzyme where the active site is located (Fig. S9). Because MraY in the YES complex is inhibited by the phage lysis protein, this opened conformation likely represents the inhibited state. The similarities between the conformational changes in MraY observed in the YES complex and upon lipid II binding in the MD analysis indicate that it is feasible for lipid II binding on the periplasmic side of the enzyme to be communicated to the active site via an alteration of the dimer interface. Accordingly, an increased mobility of TM9 on the cytoplasmic-face is also observed in the MD analysis when lipid II is bound (Fig. S8B). How the T23P and other changes that presumably activate MraY by reducing the sensitivity of the enzyme to inhibition by lipid II are not yet clear. However, electron density corresponding to the lipid is still observed at the dimer interface between MraY(T23P) protomers in the variant YES complex. Although this result may be affected by the enzyme being stuck in an inhibited state by the phage inhibitor, it suggests that T23P and other changes in MraY may affect the conformational response of the enzyme to lipid II binding rather than the binding event itself. Consistent with this possibility, tyrosine at position 21 has an altered conformation in the MraY(T23P) structure in which its hydroxyl group forms a hydrogen bond network with Y227 and K358 on the opposing monomer (Fig. S10). Substitutions within these residues were also identified in the screen for hyperactive MraY enzymes, and Y227 is in the periplasmic helix that was found to be altered in the MD analysis upon lipid II binding. Thus, alterations affecting interactions in this region may be responsible for the regulation of MraY activity and its potential modulation by lipid II binding.

MraY belongs to the polyprenyl-phosphate N-acetylhexosamine 1-phosphate transferase (PNPT) superfamily of proteins that are found in all domains of life. The superfamily includes enzymes that initiate the lipid-linked stages of many glycan polymers including O-antigens, capsules, and teichoic acids in bacteria. A well-studied example outside of bacteria is the GlcNAc-1-P-transferase (GPT) that catalyzes the first step of N-linked protein glycosylation in eukaryotes by conjugating GlcNAc to the lipid carrier dolichol phosphate (DolP) to form Dol-PP-GlcNAc^11^. In each synthesis pathway, the final lipid-linked precursor for each glycan is built on a lipid carrier that must be shared with other pathways. It would therefore not be surprising if externalized versions of the final lipid-linked precursors of many different glycan biogenesis pathways exerted feedback control on the PNPT superfamily member that initiates precursor synthesis. Such a broad utilization of this feedback regulation would provide a mechanism to efficiently distribute limiting lipid carrier molecules between competing glycan synthesis pathways in cells by matching precursor supply with utilization.

In summary, we provide evidence that the essential and broadly conserved MraY step in PG synthesis is subject to a previously unknown regulatory mechanism. Mutational and structural evidence identified the likely regulatory site on the enzyme. Importantly, this site is accessible by small molecules from the extracytoplasmic side of the membrane unlike the active site, which is in the cytoplasm. This regulatory site therefore represents an attractive new target for the development of small molecule inhibitors of MraY for potential use as antibiotics.

## Supplementary Material

Supplement 1 **Supplementary Movie 1: Movie of Lipid II binding MraY dimer**. 10 µs simulation of the top view of the MraY dimer in a mixed CG membrane. Representative simulation of two lipid II molecules (highlighted as green, gold, and pink spheres) freely entering the MraY cavity during unbiased MD simulations.

Supplement 2 **Supplementary Movie 2: Movie of C55P binding MraY dimer.** 10 µs simulation of the top view of the MraY dimer in a mixed CG membrane. One C55P molecule (highlighted as green and gold spheres) freely enters the MraY cavity during unbiased MD simulations when lipid I and lipid II are omitted.

Supplement 3 **Supplementary Figure 1. Catalytic activity is required for MraY(T23P) to suppress cell wall defects.** Ten-fold serial dilutions of cells of the indicated *P. aeruginosa* strains harboring expression plasmids producing the indicated MraY variant were plated on media with or without IPTG to induce production of MraY variants as indicated.
**Supplementary Figure 2. Cells produce MraY(WT) and MraY(T23P) to comparable levels.** (**A**) Ten-fold serial dilutions of *P. aeruginosa* cells harboring expression plasmids producing the indicated VSVG-tagged MraY were plated on media with or without inducer as indicated. (**B**) Western blot of cells expressing MraY(WT)-VSVG or MraY(T23P)-VSVG. *P. aeruginosa* cells expressing the indicated plasmid were grown to mid-log, normalized for optical density, and extracts were prepared for immunoblotting. Protein was detected using α-VSVG antibody.
**Supplementary Figure 3: Lipid I levels in cells producing MraY(WT) or MraY(T23P).** (**A**) Chemical structures of the Lipid II (LII) and Lipid I (LI) hydrolysis products detected by LCMS. Quantification of extracted ion chromatograms of the lipid I hydrolysis product for the indicated *P. aeruginosa* (**B**) and *E. coli* (**C**) strains. Three independent extractions were performed with lipid I levels quantified using the area of the peak from the extracted ion chromatogram using the Agilent software. Error bars represent SD. For MraY(T23P) vs MraY(WT) in PAO1 Δ*ponB* Δ*lpoA* P<0.05, in PAO1, MG1655, MG1655 Δ*ponA* Δ*lpoB ponB*[E313D], not significant.
**Supplementary Figure 4: Expression of** ^***Pa***^**MraY(T23P) causes a pyocin-dependent growth defect in *P. aeruginosa* due to a reduction in O-antigen production.** (**A**) Ten-fold serial dilutions of *P. aeruginosa* strains harboring expression plasmids producing the indicated MraY variant were plated on LB containing with or without IPTG to induce protein production from the plasmids. (**B**) Western blot of B-band O-antigen from *P. aeruginosa* cells expressing the MraY proteins as indicated. Image is representative of three independent experiments. (**C**) The B-band LPS from three independent replicates of sample extraction was quantified using densitometry. Error bars represent SEM, P <0.05.
**Supplementary Figure 5. Cryo-EM structure of** ^***Ec***^**MraY(T23P) in the YES complex.** Data processing was performed using cryosparc (v3.2.0). Representative movies are shown (top left) with corresponding 2D classes observed in the dataset. Arrows denote the methodology order, following several rounds of heterogeneous refinement. The number of particles sorted is shown below the densities. The masked volume of MraY (green, top right) used for particle subtraction is shown overlayed with the density (purple) of the entire YES complex. The final model is colored by resolution using the viridis color scheme. The unmodeled density at the dimer-interface is isolated for clarity and shown in a dotted box.
**Supplementary Figure 6. MraY residues contacting lipid II in the MD simulations.** Lipid II contacts with MraY residues from atomistic MD simulations. Error bars represent standard error from 5 repeats. Darker green bars represent residues altered in hyperactive variants. Dashed line at x=0.6 represents cutoff for interactions shown in Figure 4C.
**Supplementary Figure 7. Flexibility of MraY bound lipid II in the MD simulation.** All states of lipid II from 5 repeats of atomistic simulation overlaid onto the structure of MraY. Colored as in Fig. 4D.
**Supplementary Figure 8. MD analysis identifies potential conformational changes in MraY upon lipid II binding. (A)** Structure of MraY dimer in state when lipid II is bound (not shown). Residues V208 and S226 are indicated and colored purple. **(B-D)** An overlay of the structure of MraY at the end of simulations with (purple) or without (gray) lipid II present. **(B)** The structure is shown from the top, lipid II is hidden, and helices with notable differences are indicated. **(C, D)** MraY with lipid II, boxes indicate where lipid II clashes with the structure from the simulation without lipid II, indicating why the periplasmic helix 221–228 is moved apart when lipid II is bound. **(C)** is top (periplasmic) view, while **(D)** is a side view. **(E)** A boxplot of the average distance between V208 (a residue in the lipid II binding pocket) of each MraY monomer, in simulations with or without lipid II present. The data represented by each box plot is the mean distance from all time points in each of 5 repeats. **(F)** A boxplot of the average distance between S226 (a residue in the periplasmic helix above the lipid II binding site) of each MraY monomer, in simulations with or without lipid II present. The data represented by each box plot is the mean distance from all time points in each of 5 repeats.
**Supplementary Figure 9. Altered conformation of MraY dimers in the YES complex versus** ^***Aa***^**MraY.** View from the plane of the membrane. Stick representation of the α-carbon chain of ^*Ec*^MraY(T23P) (pink) structurally aligned to ^*Aa*^MraY (PDBID:4J72)(green). Molecules are aligned to the right chains in the figure. Arrows highlight the differences in ^*Aa*^MraY compared to ^*Ec*^MraY(T23P).
**Supplementary Figure 10. Comparison of MraY(WT) and MraY(T23P) structures in the YES complex.** (**A**) Overlay of densities of MraY(WT) (EMDB-29641) (green) and MraY(T23P) (purple) viewed in the plane of the membrane. (**B**) Enlarged view of the densities around the T23P mutant. Residues are shown in stick representation. Residues 21–23 are labeled for reference. (**C**) As in *B* for the wild-type complex. (**D**) Hydrogen bonding network observed in MraY(T23P) (left, purple) compared to WT (right, green) at the mutagenesis site involving Y21, Y227, and K358. (**E**) Similar to *D,* overlay of the two models highlighting the conformational differences of residue Y21.

## References

[1] May K. L. & Silhavy T. J. Making a membrane on the other side of the wall. Biochimica Et Biophysica Acta Bba - Mol Cell Biology Lipids 1862, 1386–1393 (2017). 10.1016/j.bbalip.2016.10.004 PMC5388599 27742351

[2] Silhavy T. J., Kahne D. & Walker S. The Bacterial Cell Envelope. Csh Perspect Biol 2, a000414 (2010). 10.1101/cshperspect.a000414 PMC2857177 20452953

[3] Huszczynski S. M., Lam J. S. & Khursigara C. M. The Role of Pseudomonas aeruginosa Lipopolysaccharide in Bacterial Pathogenesis and Physiology. Pathogens 9, 6 (2019). 31861540 10.3390/pathogens9010006 PMC7168646

[4] Rohs P. D. A. & Bernhardt T. G. Growth and Division of the Peptidoglycan Matrix. Annu. Rev. Microbiol. 75, 1–22 (2021). 34351794 10.1146/annurev-micro-020518-120056

[5] Piepenbreier H., Diehl A. & Fritz G. Minimal exposure of lipid II cycle intermediates triggers cell wall antibiotic resistance. Nat Commun 10, 2733 (2019). 31227716 10.1038/s41467-019-10673-4 PMC6588590

[6] Barreteau H. Quantitative high-performance liquid chromatography analysis of the pool levels of undecaprenyl phosphate and its derivatives in bacterial membranes. J Chromatogr B 877, 213–220 (2009). 10.1016/j.jchromb.2008.12.010 19110475

[7] Hartley M. D. & Imperiali B. At the membrane frontier: A prospectus on the remarkable evolutionary conservation of polyprenols and polyprenyl-phosphates. Arch Biochem Biophys 517, 83–97 (2012). 22093697 10.1016/j.abb.2011.10.018 PMC3253937

[8] Jorgenson M. A. & Young K. D. Interrupting Biosynthesis of O Antigen or the Lipopolysaccharide Core Produces Morphological Defects in Escherichia coli by Sequestering Undecaprenyl Phosphate. J Bacteriol 198, 3070–3079 (2016). 27573014 10.1128/JB.00550-16 PMC5075036

[9] Jorgenson M. A., Kannan S., Laubacher M. E. & Young K. D. Dead‐end intermediates in the enterobacterial common antigen pathway induce morphological defects in Escherichia coli by competing for undecaprenyl phosphate. Mol Microbiol 100, 1–14 (2016). 26593043 10.1111/mmi.13284 PMC4845916

[10] D’Elia M. A. Lesions in teichoic acid biosynthesis in Staphylococcus aureus lead to a lethal gain of function in the otherwise dispensable pathway. J Bacteriol 188, 4183–9 (2006). 16740924 10.1128/JB.00197-06 PMC1482942

[11] Lehrman M. A. Biosynthesis of N -acetylglucosamine-P-P-dolichol, the committed step of asparagine-linked oligosaccharide assembly. Glycobiology 1, 553–562 (1991). 1668306 10.1093/glycob/1.6.553

[12] Sham L.-T. MurJ is the flippase of lipid-linked precursors for peptidoglycan biogenesis. Science 345, 220–222 (2014). 25013077 10.1126/science.1254522 PMC4163187

[13] Sauvage E., Kerff F., Terrak M., Ayala J. A. & Charlier P. The penicillin-binding proteins: structure and role in peptidoglycan biosynthesis. Fems Microbiol Rev 32, 234–258 (2008). 18266856 10.1111/j.1574-6976.2008.00105.x

[14] Zhao H., Patel V., Helmann J. D. & Dörr T. Don’t let sleeping dogmas lie: New views of peptidoglycan synthesis and its regulation. Mol Microbiol 106, 847–860 (2017). 28975672 10.1111/mmi.13853 PMC5720918

[15] Taguchi A. FtsW is a peptidoglycan polymerase that is functional only in complex with its cognate penicillin-binding protein. Nat Microbiol 4, 587–594 (2019). 30692671 10.1038/s41564-018-0345-x PMC6430707

[16] Meeske A. J. SEDS proteins are a widespread family of bacterial cell wall polymerases. Nature 537, 634–638 (2016). 27525505 10.1038/nature19331 PMC5161649

[17] Rohs P. D. A. A central role for PBP2 in the activation of peptidoglycan polymerization by the bacterial cell elongation machinery. Plos Genet 14, e1007726 (2018). 30335755 10.1371/journal.pgen.1007726 PMC6207328

[18] Greene N. G., Fumeaux C. & Bernhardt T. G. Conserved mechanism of cell-wall synthase regulation revealed by the identification of a new PBP activator in Pseudomonas aeruginosa. Proc National Acad Sci 115, 3150–3155 (2018). 10.1073/pnas.1717925115 PMC5866570 29507210

[19] Paradis-Bleau C. Lipoprotein Cofactors Located in the Outer Membrane Activate Bacterial Cell Wall Polymerases. Cell 143, 1110–1120 (2010). 21183074 10.1016/j.cell.2010.11.037 PMC3085243

[20] Sardis M. F., Bohrhunter J. L., Greene N. G. & Bernhardt T. G. The LpoA activator is required to stimulate the peptidoglycan polymerase activity of its cognate cell wall synthase PBP1a. Proc National Acad Sci 118, e2108894118 (2021). 10.1073/pnas.2108894118 PMC8536351 34429361

[21] Typas A. Regulation of Peptidoglycan Synthesis by Outer-Membrane Proteins. Cell 143, 1097–1109 (2010). 21183073 10.1016/j.cell.2010.11.038 PMC3060616

[22] Markovski M. Cofactor bypass variants reveal a conformational control mechanism governing cell wall polymerase activity. Proc National Acad Sci 113, 4788–4793 (2016). 10.1073/pnas.1524538113 PMC4855605 27071112

[23] Köhler T., Donner V. & Delden C. van. Lipopolysaccharide as Shield and Receptor for R-Pyocin-Mediated Killing in Pseudomonas aeruginosa. J Bacteriol 192, 1921–1928 (2010). 20118263 10.1128/JB.01459-09 PMC2838038

[24] Ge P. Action of a minimal contractile bactericidal nanomachine. Nature 580, 658–662 (2020). 32350467 10.1038/s41586-020-2186-z PMC7513463

[25] Penterman J. Rapid Evolution of Culture-Impaired Bacteria during Adaptation to Biofilm Growth. Cell Reports 6, 293–300 (2014). 24412364 10.1016/j.celrep.2013.12.019 PMC3941072

[26] Chung B. C. Crystal Structure of MraY, an Essential Membrane Enzyme for Bacterial Cell Wall Synthesis. Science 341, 1012–1016 (2013). 23990562 10.1126/science.1236501 PMC3906829

[27] Varadi M. AlphaFold Protein Structure Database: massively expanding the structural coverage of protein-sequence space with high-accuracy models. Nucleic Acids Res 50, D439–D444 (2022). 34791371 10.1093/nar/gkab1061 PMC8728224

[28] Jumper J. Highly accurate protein structure prediction with AlphaFold. Nature 596, 583–589 (2021). 34265844 10.1038/s41586-021-03819-2 PMC8371605

[29] Hakulinen J. K. MraY–antibiotic complex reveals details of tunicamycin mode of action. Nat Chem Biol 13, 265–267 (2017). 28068312 10.1038/nchembio.2270

[30] Oluwole A. O. Peptidoglycan biosynthesis is driven by lipid transfer along enzyme-substrate affinity gradients. Nat Commun 13, 2278 (2022). 35477938 10.1038/s41467-022-29836-x PMC9046198

[31] Orta A. K. The mechanism of the phage-encoded protein antibiotic from ΦX174. Science 381, (2023). 10.1126/science.adg9091 PMC12747129 37440661

