# Supplementary material for: A feedback control mechanism governs the synthesis of lipid-linked precursors of the bacterial cell wall
Source: bioRxiv. 2023 Aug 1:2023.08.01.551478. Preprint. [Version 1] doi: 10.1101/2023.08.01.551478 (PMC10418202; doi:10.1101/2023.08.01.551478)
Supplement: Supplement 3 — Supplementary Figure 1. Catalytic activity is required for MraY(T23P) to suppress cell wall defects. Ten-fold serial dilutions of cells of the indicated P. aeruginosa strains harboring expression plasmids producing the indicated MraY variant were plated on media with or without IPTG to induce production of MraY variants as indicated. Supplementary Figure 2. Cells produce MraY(WT) and MraY(T23P) to comparable levels. (A) Ten-fold serial dilutions of P. aeruginosa cells harboring expression plasmids producing the indicated VSVG-tagged MraY were plated on media with or without inducer as indicated. (B) Western blot of cells expressing MraY(WT)-VSVG or MraY(T23P)-VSVG. P. aeruginosa cells expressing the indicated plasmid were grown to mid-log, normalized for optical density, and extracts were prepared for immunoblotting. Protein was detected using α-VSVG antibody. Supplementary Figure 3: Lipid I levels in cells producing MraY(WT) or MraY(T23P). (A) Chemical structures of the Lipid II (LII) and Lipid I (LI) hydrolysis products detected by LCMS. Quantification of extracted ion chromatograms of the lipid I hydrolysis product for the indicated P. aeruginosa (B) and E. coli (C) strains. Three independent extractions were performed with lipid I levels quantified using the area of the peak from the extracted ion chromatogram using the Agilent software. Error bars represent SD. For MraY(T23P) vs MraY(WT) in PAO1 ΔponB ΔlpoA P<0.05, in PAO1, MG1655, MG1655 ΔponA ΔlpoB ponB[E313D], not significant. Supplementary Figure 4: Expression of PaMraY(T23P) causes a pyocin-dependent growth defect in P. aeruginosa due to a reduction in O-antigen production. (A) Ten-fold serial dilutions of P. aeruginosa strains harboring expression plasmids producing the indicated MraY variant were plated on LB containing with or without IPTG to induce protein production from the plasmids. (B) Western blot of B-band O-antigen from P. aeruginosa cells expressing the MraY proteins as indicated. Image is repr [file NIHPP2023.08.01.551478v1-supplement-3.pdf]

bioRxiv preprint doi: https://doi.org/10.1101/2023.08.01.551478; this version posted August 1, 2023. The copyright holder for this preprint (which was not certified by peer review) is the author/funder, who has granted bioRxiv a license to display the preprint in perpetuity. It is made available under aCC-BY 4.0 International license.

Supplementary Figure 1

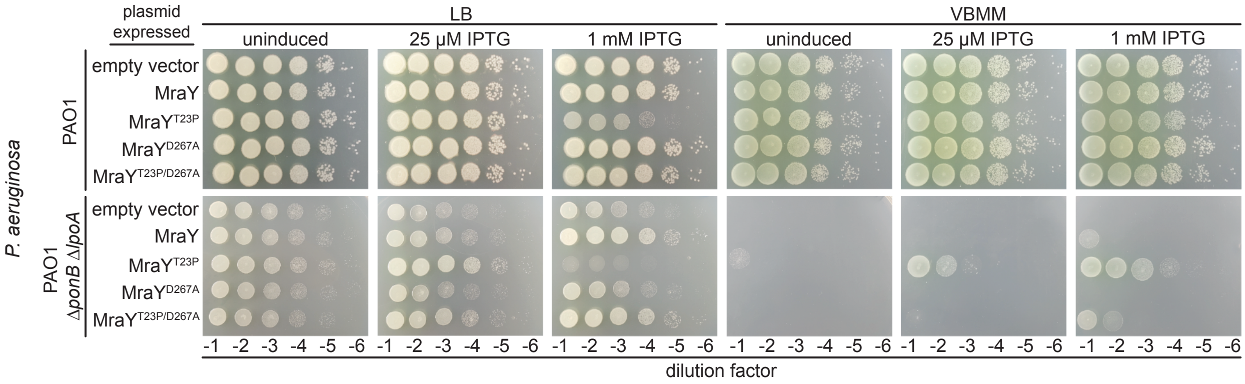

Supplementary Figure 1. Catalytic activity is required for MraY(T23P) to suppress cell wall defects. Ten-fold serial dilutions of cells of the indicated *P. aeruginosa* strains harboring expression plasmids producing the indicated MraY variant were plated on media with or without IPTG to induce production of MraY variants as indicated.

bioRxiv preprint doi: https://doi.org/10.1101/2023.08.01.551478; this version posted August 1, 2023. The copyright holder for this preprint (which was not certified by peer review) is the author/funder, who has granted bioRxiv a license to display the preprint in perpetuity. It is made available under aCC-BY 4.0 International license.

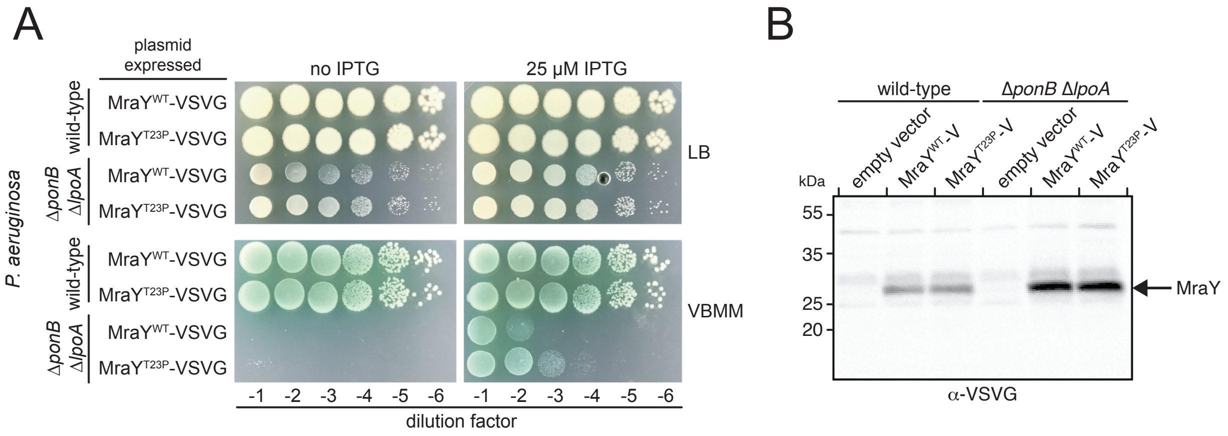

Supplementary Figure 2. Cells produce MraY(WT) and MraY(T23P) to comparable levels. (**A**) Ten-fold serial dilutions of *P. aeruginosa* cells harboring expression plasmids producing the indicated VSVG-tagged MraY were plated on media with or without inducer as indicated. (**B**) Western blot of cells expressing MraY(WT)-VSVG or MraY(T23P)-VSVG. *P. aeruginosa* cells expressing the indicated plasmid were grown to mid-log, normalized for optical density, and extracts were prepared for immunoblotting. Protein was detected using α-VSVG antibody.

bioRxiv preprint doi: https://doi.org/10.1101/2023.08.01.551478; this version posted August 1, 2023. The copyright holder for this preprint (which was not certified by peer review) is the author/funder, who has granted bioRxiv a license to display the preprint in perpetuity. It is made available under aCC-BY 4.0 International license.

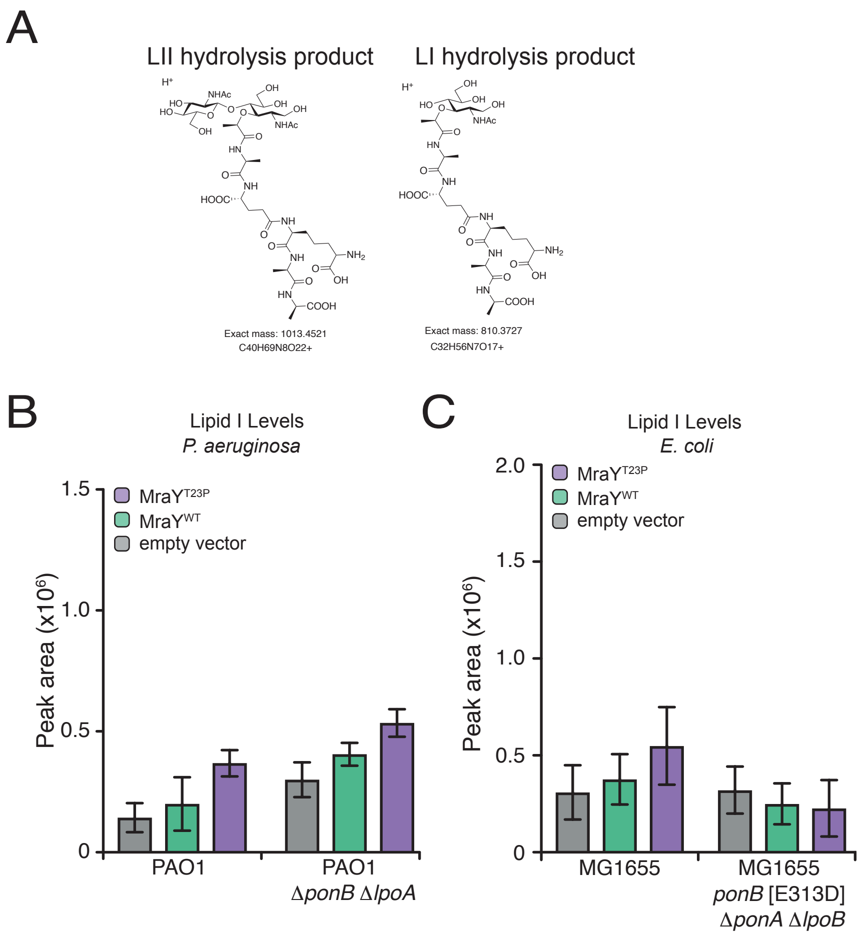

Supplementary Figure 3: Lipid I levels in cells producing MraY(WT) or MraY(T23P). (**A**) Chemical structures of the Lipid II (LII) and Lipid I (LI) hydrolysis products detected by LCMS. Quantification of extracted ion chromatograms of the lipid I hydrolysis product for the indicated *P. aeruginosa* (**B**) and *E. coli* (**C**) strains. Three independent extractions were performed with lipid I levels quantified using the area of the peak from the extracted ion chromatogram using the Agilent software. Error bars represent SD. For MraY(T23P) vs MraY(WT) in PAO1 Δ*ponB* Δ*lpoA* $P<0.05$, in PAO1, MG1655, MG1655 Δ*ponA* Δ*lpoB* ponB[E313D], not significant.

bioRxiv preprint doi: https://doi.org/10.1101/2023.08.01.551478; this version posted August 1, 2023. The copyright holder for this preprint (which was not certified by peer review) is the author/funder, who has granted bioRxiv a license to display the preprint in perpetuity. It is made available under aCC-BY 4.0 International license.

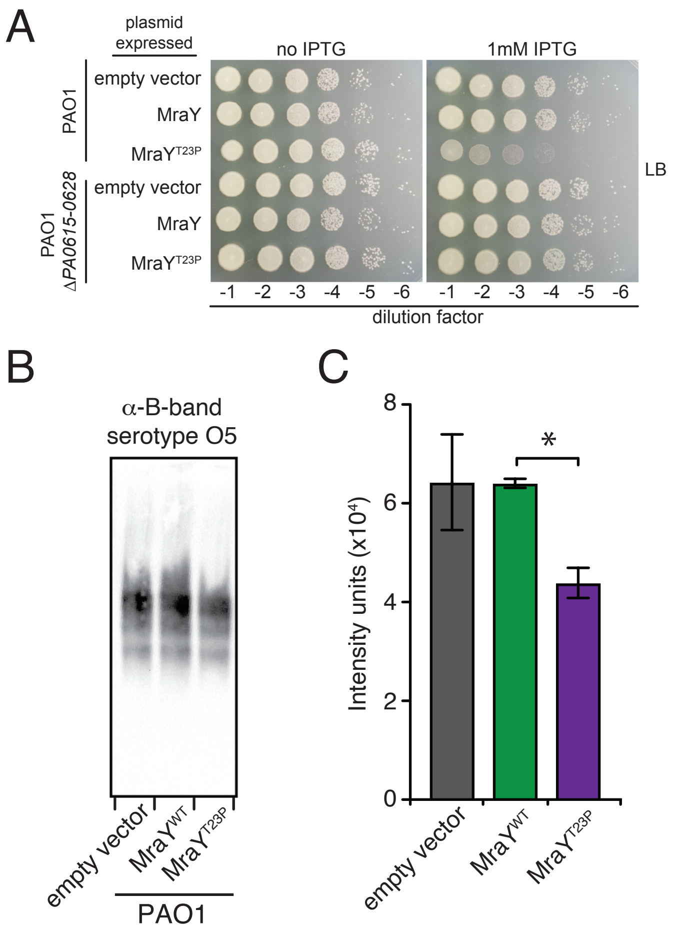

Supplementary Figure 4: Expression of ^{Pa}MraY(T23P) causes a pyocin-dependent growth defect in *P. aeruginosa* due to a reduction in O-antigen production. (**A**) Ten-fold serial dilutions of *P. aeruginosa* strains harboring expression plasmids producing the indicated MraY variant were plated on LB containing with or without IPTG to induce protein production from the plasmids. (**B**) Western blot of B-band O-antigen from *P. aeruginosa* cells expressing the MraY proteins as indicated. Image is representative of three independent experiments. (**C**) The B-band LPS from three independent replicates of sample extraction was quantified using densitometry. Error bars represent SEM, $P < 0.05$.

bioRxiv preprint doi: https://doi.org/10.1101/2023.08.01.551478; this version posted August 1, 2023. The copyright holder for this preprint (which was not certified by peer review) is the author/funder, who has granted bioRxiv a license to display the preprint in perpetuity. It is made available under aCC-BY 4.0 International license.

Supplementary Figure 5

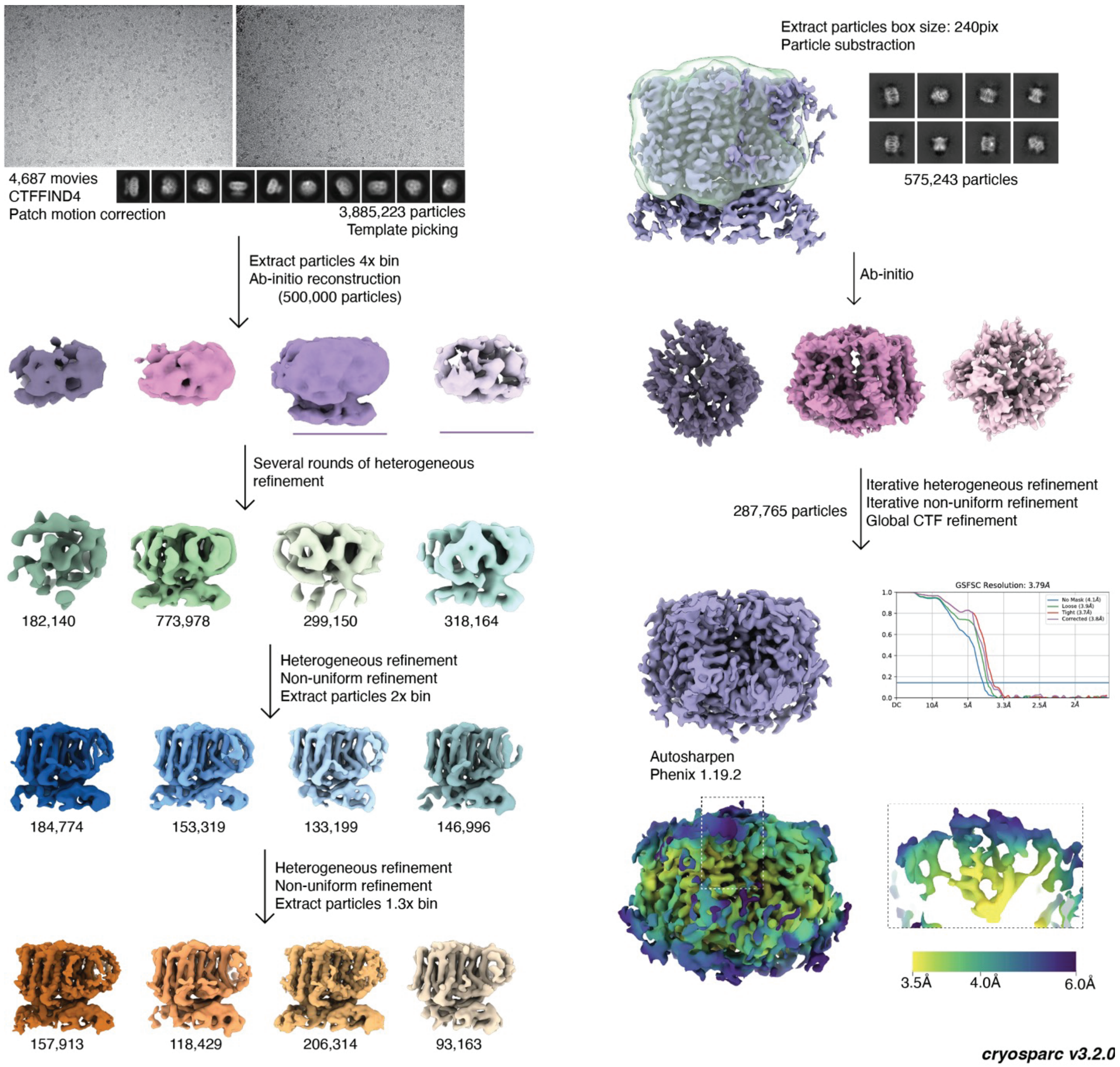

Supplementary Figure 5. Cryo-EM structure of ^{Ec}MraY(T23P) in the YES complex. Data processing was performed using cryosparc (v3.2.0). Representative movies are shown (top left) with corresponding 2D classes observed in the dataset. Arrows denote the methodology order, following several rounds of heterogeneous refinement. The number of particles sorted is shown below the densities. The masked volume of MraY (green, top right) used for particle subtraction is shown overlayed with the density (purple) of the entire YES complex. The final model is colored by resolution using the viridis color scheme. The unmodeled density at the dimer-interface is isolated for clarity and shown in a dotted box.

bioRxiv preprint doi: https://doi.org/10.1101/2023.08.01.551478; this version posted August 1, 2023. The copyright holder for this preprint (which was not certified by peer review) is the author/funder, who has granted bioRxiv a license to display the preprint in perpetuity. It is made available under aCC-BY 4.0 International license.

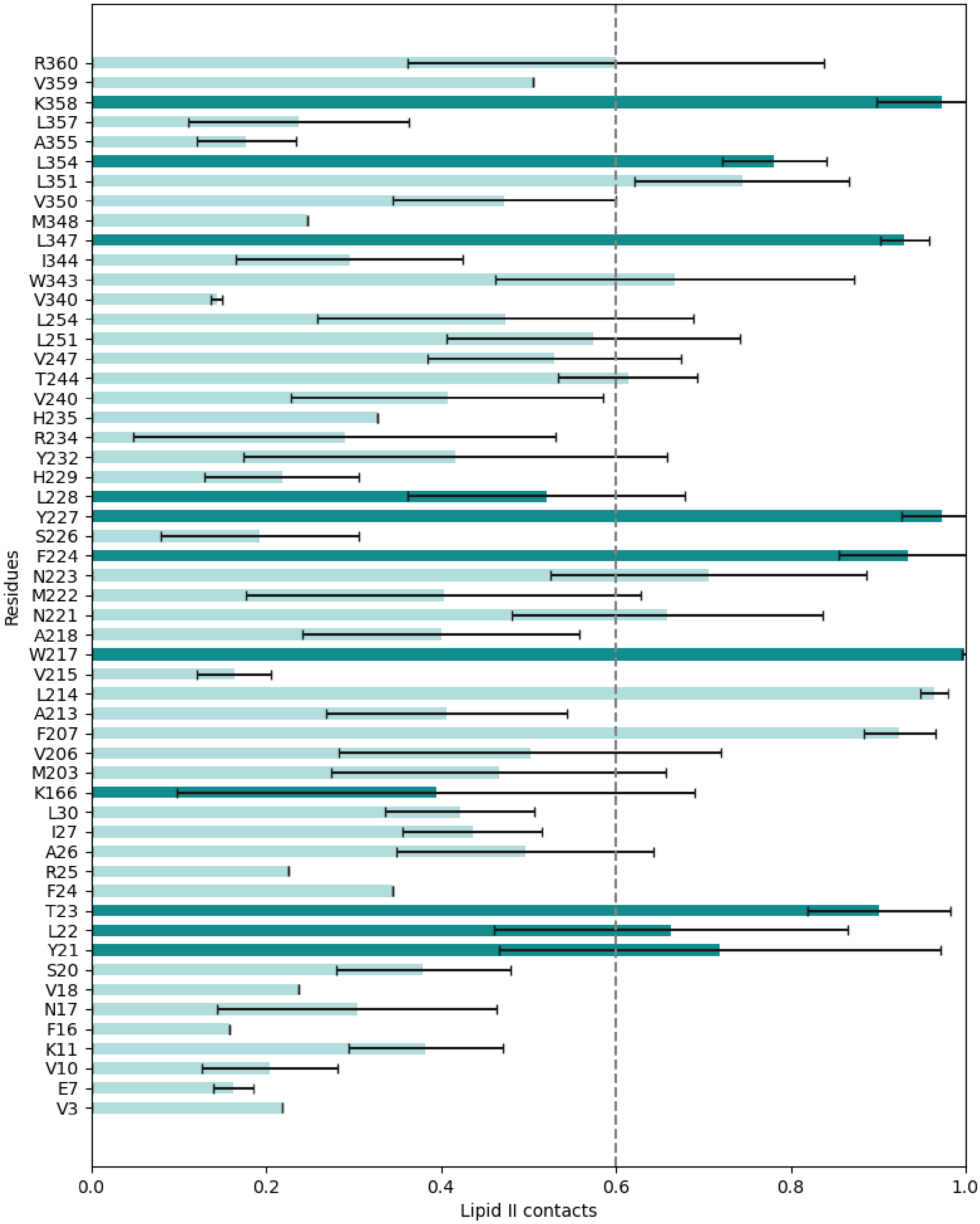

Supplementary Figure 6. MraY residues contacting lipid II in the MD simulations. Lipid II contacts with MraY residues from atomistic MD simulations. Error bars represent standard error from 5 repeats. Darker green bars represent residues altered in hyperactive variants. Dashed line at x=0.6 represents cutoff for interactions shown in Figure 4C.

bioRxiv preprint doi: https://doi.org/10.1101/2023.08.01.551478; this version posted August 1, 2023. The copyright holder for this preprint (which was not certified by peer review) is the author/funder, who has granted bioRxiv a license to display the preprint in perpetuity. It is made available under aCC-BY 4.0 International license.

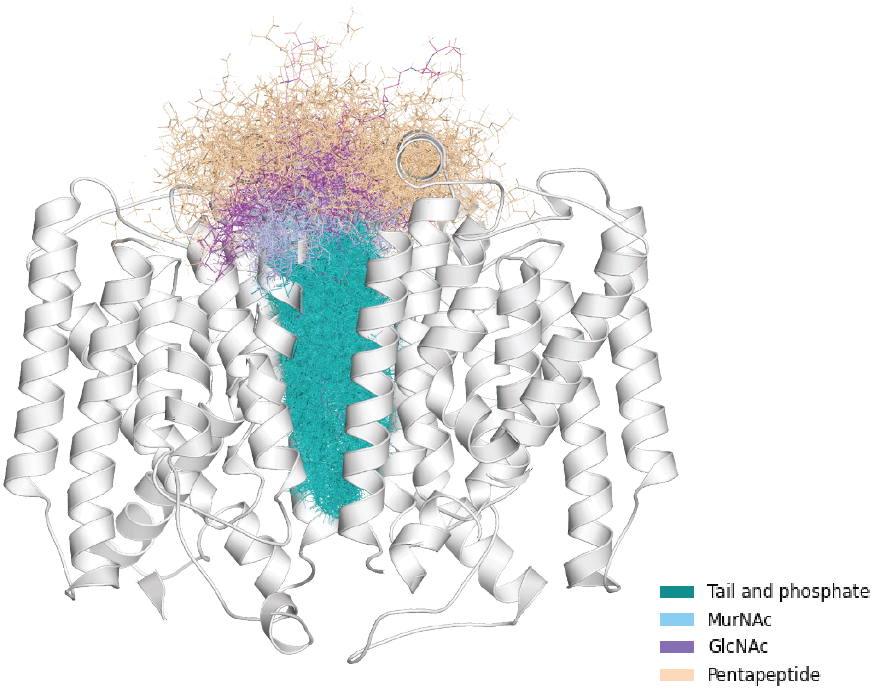

Supplementary Figure 7. Flexibility of MraY bound lipid II in the MD simulation. All states of lipid II from 5 repeats of atomistic simulation overlaid onto the structure of MraY. Colored as in Fig. 4D.

bioRxiv preprint doi: https://doi.org/10.1101/2023.08.01.551478; this version posted August 1, 2023. The copyright holder for this preprint (which was not certified by peer review) is the author/funder, who has granted bioRxiv a license to display the preprint in perpetuity. It is made available under aCC-BY 4.0 International license.

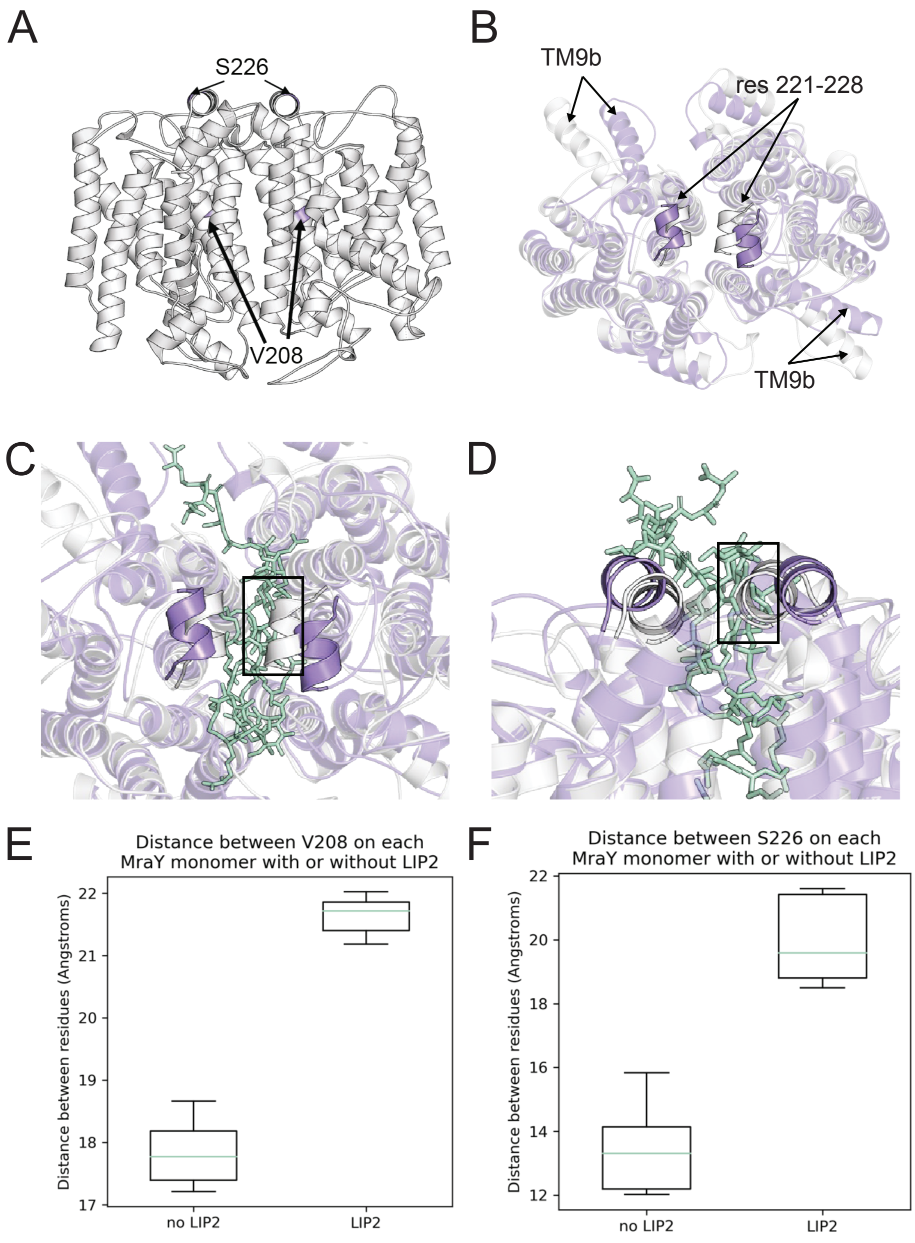

Supplementary Figure 8. MD analysis identifies potential conformational changes in MraY upon lipid II binding. (**A**) Structure of MraY dimer in state when lipid II is bound (not shown). Residues V208 and S226 are indicated and colored purple. (**B-D**) An overlay of the structure of MraY at the end of simulations with (purple) or without (gray) lipid II present. (**B**) The structure is shown from the top, lipid II is hidden, and helices with notable differences are indicated. (**C, D**) MraY with lipid II, boxes indicate where lipid II clashes with the structure from the simulation without lipid II, indicating why the periplasmic helix 221-228 is moved apart when lipid II is bound. (**C**) is top (periplasmic) view, while (**D**) is a side view. (**E**) A boxplot of the average distance between V208 (a residue in the lipid II binding pocket) of each MraY monomer, in simulations with or without lipid II present. The data represented by each box plot is the mean distance from all time points in each of 5 repeats. (**F**) A boxplot of the average distance between S226 (a residue in the periplasmic helix above the lipid II binding site) of each MraY monomer, in simulations with or without lipid II present. The data represented by each box plot is the mean distance from all time points in each of 5 repeats.

bioRxiv preprint doi: https://doi.org/10.1101/2023.08.01.551478; this version posted August 1, 2023. The copyright holder for this preprint (which was not certified by peer review) is the author/funder, who has granted bioRxiv a license to display the preprint in perpetuity. It is made available under aCC-BY 4.0 International license.

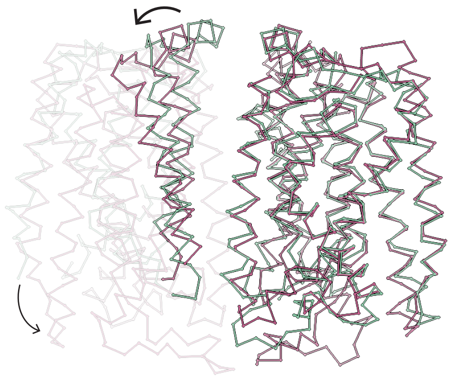

Supplementary Figure 9. Altered conformation of MraY dimers in the YES complex versus ^{Aa}MraY. View from the plane of the membrane. Stick representation of the α-carbon chain of ^{Ec}MraY(T23P) (pink) structurally aligned to ^{Aa}MraY (PDBID:4J72)(green). Molecules are aligned to the right chains in the figure. Arrows highlight the differences in ^{Aa}MraY compared to ^{Ec}MraY(T23P).

bioRxiv preprint doi: https://doi.org/10.1101/2023.08.01.551478; this version posted August 1, 2023. The copyright holder for this preprint (which was not certified by peer review) is the author/funder, who has granted bioRxiv a license to display the preprint in perpetuity. It is made available under aCC-BY 4.0 International license.

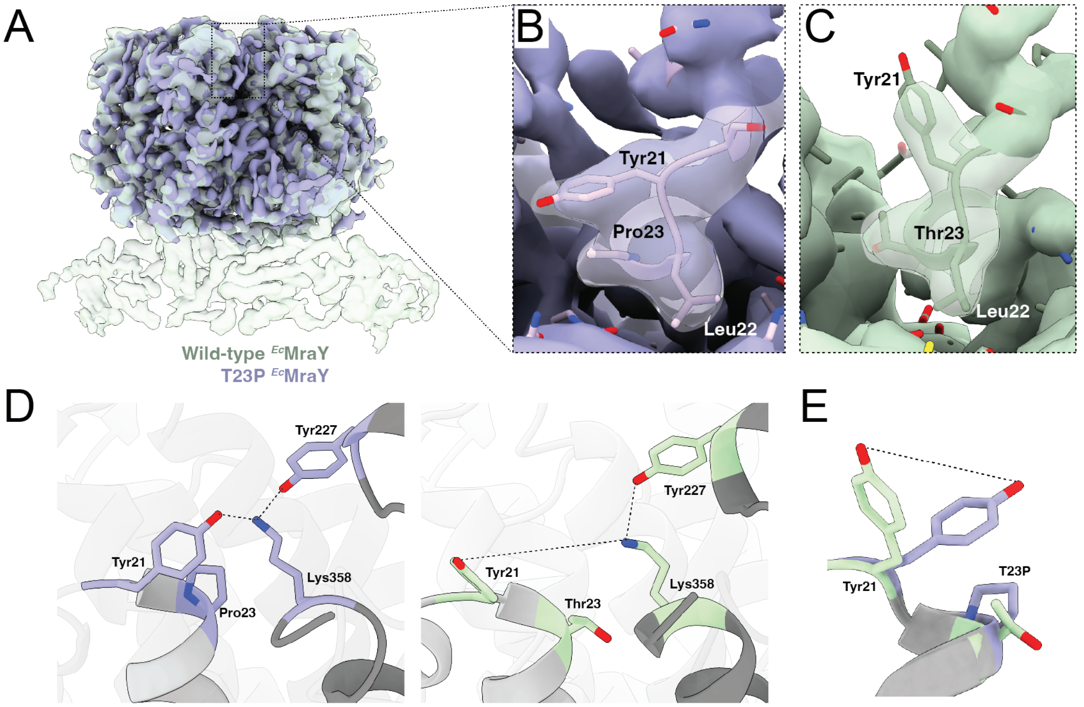

Supplementary Figure 10. Comparison of MraY(WT) and MraY(T23P) structures in the YES complex. (**A**) Overlay of densities of MraY(WT) (EMDB-29641) (green) and MraY(T23P) (purple) viewed in the plane of the membrane. (**B**) Enlarged view of the densities around the T23P mutant. Residues are shown in stick representation. Residues 21-23 are labeled for reference. (**C**) As in **B** for the wild-type complex. (**D**) Hydrogen bonding network observed in MraY(T23P) (left, purple) compared to WT (right, green) at the mutagenesis site involving Y21, Y227, and K358. (**E**) Similar to **D**, overlay of the two models highlighting the conformational differences of residue Y21.

bioRxiv preprint doi: https://doi.org/10.1101/2023.08.01.551478; this version posted August 1, 2023. The copyright holder for this preprint (which was not certified by peer review) is the author/funder, who has granted bioRxiv a license to display the preprint in perpetuity. It is made available under aCC-BY 4.0 International license.

Supplementary Material for:

A feedback control mechanism governs the synthesis of lipid-linked precursors of the bacterial cell wall

Authors: Lindsey S. Marmont[1,2], Anna K. Orta[3], Robin A. Corey[4], David Sychantha[2], Ana Fernández Galliano[2], Yancheng E. Li[3], Becca W.A. Baileeves[5], Neil G. Greene[1,+], Phillip J. Stansfeld[5], William M. Clemons, Jr.[3], and Thomas G. Bernhardt[1,6,*]

Affiliations: [1]Department of Microbiology, Blavatnik Institute, Harvard Medical School, Boston, MA 02115.
[2]M.G. DeGroote Institute for Infectious Disease Research, David Braley Centre for Antibiotic Discovery, Department of Biochemistry and Biomedical Sciences, McMaster University, Hamilton, Canada.
[3]Division of Chemistry and Chemical Engineering, California Institute of Technology, Pasadena, California, USA
[4]School of Physiology, Pharmacology, and Neuroscience, University of Bristol, Bristol, UK
[5]School of Life Sciences and Department of Chemistry, University of Warwick, Warwick, UK
[6]Howard Hughes Medical Institute, Boston, United States
[+]Present address: Biology Department, The University of Rhode Island, Rhode Island, United States.

***To whom correspondence should be addressed**.
Thomas G. Bernhardt, Ph.D.
Harvard Medical School
Department of Microbiology
Boston, Massachusetts 02115
e-mail: thomas_bernhardt@hms.harvard.edu

bioRxiv preprint doi: https://doi.org/10.1101/2023.08.01.551478; this version posted August 1, 2023. The copyright holder for this preprint (which was not certified by peer review) is the author/funder, who has granted bioRxiv a license to display the preprint in perpetuity. It is made available under aCC-BY 4.0 International license.

SI Table 1. List of suppressing MraY variants

Residue	Changed to
K14	E
Y21	H
L22	P
T23	A, P
L31	P
L160	R
M164	R
K166	E
Y217	H
G220	S
F224	S
A225	V
Y227	C
L228	P
G248	C
Q286	R
V289	M
I339	V
V347	M
L354	P
K358	M

SI Table 2: Cryo-EM data collection, refinement, and validation statistics

	***Ec*MraY** (EMDB- EMD-41373) (PDB 8TLU)
**Data collection and processing**	
Microscope	FEI Titan Krios
Magnification	105,000
Voltage (kV)	300
Electron exposure (e–/Å^2)	60
Defocus range (μm)	-1 to -2.5
Pixel size (Å)	0.832
Symmetry imposed	C1
Initial particle images (no.)	3,885,223
Final particle images (no.)	287,765
Map resolution (Å) FSC threshold: 0.143	3.8
**Refinement**	
Software	PHENIX 1.19.2
Initial model used (PDB code)	8G01
Resolution of unmasked reconstructions (Å, FSC=0.5)	4.1

bioRxiv preprint doi: https://doi.org/10.1101/2023.08.01.551478; this version posted August 1, 2023. The copyright holder for this preprint (which was not certified by peer review) is the author/funder, who has granted bioRxiv a license to display the preprint in perpetuity. It is made available under aCC-BY 4.0 International license.

Resolution of masked reconstructions (Å, FSC=0.5)	3.8
Correlation coefficient (CC_{mask})	0.76
Model composition	
Atoms (Hydrogens)	11180 (5703)
Protein residues	698
Ligands	0
B factors (Å^2) (min/max/mean)	
Protein	20.34/79.77/41.69
Ligand	-
R.m.s. deviations	
Bond lengths (Å)	0.003 (0)
Bond angles (°)	0.536 (0)
**Validation**	
MolProbity score	1.43
Clashscore	7.87
Poor rotamers (%)	0
Ramachandran plot	
Favored (%)	98.40
Allowed (%)	1.60
Disallowed (%)	0

SI Table 3. Oligonucleotide primers used in this study

Primer	Sequence (5'-3')*
oNG338	ATGCGAATTC**GAGGAGGATACAT**ATGCTCCTGCTGCTGGCC
oNG339	AATTCCCGGGTCAACGCAGCTTCAAGGTGG
oLSM301	CAGCAGGAGCATGGATGACCCCCCAGGGCCT
oLSM302	GGGGTCATCCATGCTCCTGCTGCTGGCCGAAT
oLSM303	GCAGCCTAGGTTAATCAACGCAGCTTCAAGGTGGCGA
oLSM304	GAAGCTGCGTTGATTAACCTAGGCTGCTGCCACCG
oLSM311	CAGCCAAACTAACATATGTATCCTCCTCGAATTCCTAGCTAG
oLSM312	TCGAGGAGGATACATATGTTAGTTTGGCTGGCCGAACATTTG
oLSM313	GGATCCCCGGGTTAACGTACCTTCAGCGTTGCCAGA
oLSM314	TGAAGGTACGTTAACCCGGGGATCCTCTAGAGTC
oLSM315	GTCTTTTCCTATCTGCCGTTTCGCGCC
oLSM316	GCGCGAAACGGCAGATAGGAAAAGACGTTAAA
oLSM317	ATTCGAGGAGGATACATATGCTCCTGCTGCTGGCCGAATA
oLSM318	GCAGGAGCATATGTATCCTCCTCGAATTCCTAGCTAG
oLSM368	ATGTATCCTCCTCTCTAGAGGGGAATTGTTATCCGCTCA
oLSM372	CAATTCCCCTCTAGAGAGGAGGATACATATGCTCCTGCT
oLSM373	CGGTGATAAGCTTTCAACGCAGCTTCAAGGTGGCGA
oLSM374	GAAGCTGCGTTGAAAGCTTATCACCGATACGCGAGCG
oLSM375	CAATTCCCCTCTAGAGAGGAGGATACATATGTTAGTTTGGCTG
oLSM376	GTGATAAGCTTTTAACGTACCTTCAGCGTTGCCAGA
oLSM377	CAACGCTGAAGGTACGTTAAAAGCTTATCACCGATACGCGAGCG
oLSM404	GGTCCTATACAGATATTGAAATGAATAGATTAGGAAAATGACCCGGGGATCCTCTAGA
oLSM405	TTTTCCTAATCTATTCATTTCAATATCTGTATAGGACCCACGCAGCTTCAAGGTGGCGA
oLSM460	TCTTCATGGGCGCCGTCGGCGCGCT

bioRxiv preprint doi: https://doi.org/10.1101/2023.08.01.551478; this version posted August 1, 2023. The copyright holder for this preprint (which was not certified by peer review) is the author/funder, who has granted bioRxiv a license to display the preprint in perpetuity. It is made available under aCC-BY 4.0 International license.

oLSM461	AGCGCGCCGACGGCGCCCATGAAGA
48	CATTCATGTTGTTGCTCAGGTCGCAGAC
34	CTCCTTAAGATTCTTGTACGACGG
2325	AGAGGCCCCAAGGGGTTATGCT
556	CCAGGCTTTACACTTTATGCTTCCG
557	CTGCGCAACTGTTGGGAAGG

*Restriction site underlined

SI Table 4. Bacterial strains used in this study

Strain	Description*	Reference
E. coli		
DH5α	Host strain for plasmid cloning	Invitrogen
BL21(DE3)	Expression strain for protein production	[1]
CAM333	C43(DE3) Δ*ponB*, Δ*pbpC*, Δ*mtgA*	[2]
LSM9	C43(DE3) Δ*pbpC*, Δ*mtgA fhuA-*	This study
Δ*slyD* BL21(DE3)		[3]
MG1655	Wild-type	[4]
MM119	Δ*ponA ponB(*E313D) Δ*lpoB*	[5]
P. aeruginosa		
PAO1	Wild-type	S. Lory
PA686	Δ*ponB* Δ*lpoA*	[6]
PA760	Δ*ponB* Δ*lpoA mraY*(T23P)	This study
PA662	Δ*PA0615–0628*; R2 pyocin deletion	S. Lory

SI Table 5. Plasmids used in this study

Plasmid	Description*	Reference
pETDuet-1	IPTG-inducible protein expression vector containing two multiple cloning sites; pBR322-derived ColE1 replicon; $Carb^R$	Novagen
pETDuet-FLAG	Modified pETDuet-1 containing a N-terminal His_6-SUMO-FLAG tag at the first insertion site and C-terminal His_6 tag at the second site; $Carb^R$	[7]
pCOLADuet-1	IPTG-inducible protein expression vector containing two multiple cloning sites; COLA replicon, Kan^R	Novagen
pET22b-$SlyD_{1-154}$	construct expresses *E. coli* SlyD, modified by the removal of the flexible C-terminus	[3]
pRSFDuet*Ec*MraY-E_{ID21}	contains the ID21 isoform of protein E, along with a wild-type EcMraY to prevent cell lysis from the overexpression of protein E.	[3]
pAM174	Encodes arabinose-inducible Ulp1[L403-K621] protease; Cm^R	[2]
pPBP4	*S. aureus* His_6-PBP4 expression vector; Kan^R	[8]
pPSV38	*P. aeruginosa* expression vector containing IPTG-inducible P_{lacUV5} promoter; $Gent^R$	[9]
pRY47	*E. coli* expression vector lacIq P_{lac}::empty; pBR/colE1; Cm^R	[10]
pNG93	*P. aeruginosa* P_{lacUV5}-*Pa-mraY* expression vector (pPSV38); $Gent^R$	This study
pNG102	*P. aeruginosa* P_{lacUV5}-*Pa-mraY*^{T23P} expression vector (pPSV38); $Gent^R$	This study
pLSM116	pCOLADuet containing His_6-Sumo-FLAG-MraY; pT7; Kan^R	This study
pLSM117	pCOLADuet containing His_6-Sumo-FLAG-MraY^{T23P}; pT7; Kan^R	This study
pLSM124	*P. aeruginosa* P_{lacUV5}-*Ec-mraY* expression vector (pPSV38); $Gent^R$	This study
pLSM125	*P. aeruginosa* P_{lacUV5}-*Ec-mraY*^{T23P} expression vector (pPSV38); $Gent^R$	This study
pLSM141	*E. coli* P_{lac}-*Pa-mraY* expression vector (pRY47); Cm^R	This study
pLSM142	*E. coli* P_{lac}-*Pa-mraY*^{T23P} expression vector (pRY47); Cm^R	This study

bioRxiv preprint doi: https://doi.org/10.1101/2023.08.01.551478; this version posted August 1, 2023. The copyright holder for this preprint (which was not certified by peer review) is the author/funder, who has granted bioRxiv a license to display the preprint in perpetuity. It is made available under aCC-BY 4.0 International license.

pLSM143	*E. coli* P_{lac}-*Ec-mraY* expression vector (pRY47); Cm^R	This study
pLSM144	*E. coli* P_{lac}-*Ec-mraY*^{T23P} expression vector (pRY47); Cm^R	This study
pLSM151	*P. aeruginosa* P_{lacUV5}-*Pa-mraY*(L22P) expression vector (pPSV38); Gent^R	This study
pLSM152	*P. aeruginosa* P_{lacUV5}-*Pa-mraY*(M164R) expression vector (pPSV38); Gent^R	This study
pLSM153	*P. aeruginosa* P_{lacUV5}-*Pa-mraY*(K358M) expression vector (pPSV38); Gent^R	This study
pLSM154	*P. aeruginosa* P_{lacUV5}-*Pa-mraY*(G248C) expression vector (pPSV38); Gent^R	This study
pLSM155	*P. aeruginosa* P_{lacUV5}-*Pa-mraY*(L31P) expression vector (pPSV38); Gent^R	This study
pLSM156	*P. aeruginosa* P_{lacUV5}-*Pa-mraY*(Y227C) expression vector (pPSV38); Gent^R	This study
pLSM157	*P. aeruginosa* P_{lacUV5}-*Pa-mraY*(T23A) expression vector (pPSV38); Gent^R	This study
pLSM158	*P. aeruginosa* P_{lacUV5}-*Pa-mraY*(L354P) expression vector (pPSV38); Gent^R	This study
pLSM159	*P. aeruginosa* P_{lacUV5}-*Pa-mraY*(I339V) expression vector (pPSV38); Gent^R	This study
pLSM160	*P. aeruginosa* P_{lacUV5}-*Pa-mraY*(Y21H) expression vector (pPSV38); Gent^R	This study
pLSM161	*P. aeruginosa* P_{lacUV5}-*Pa-mraY*(L228P) expression vector (pPSV38); Gent^R	This study
pLSM162	*P. aeruginosa* P_{lacUV5}-*Pa-mraY*(Y217H) expression vector (pPSV38); Gent^R	This study
pLSM163	*P. aeruginosa* P_{lacUV5}-*Pa-mraY*(G220S) expression vector (pPSV38); Gent^R	This study
pLSM164	*P. aeruginosa* P_{lacUV5}-*Pa-mraY*(V289M) expression vector (pPSV38); Gent^R	This study
pLSM165	*P. aeruginosa* P_{lacUV5}-*Pa-mraY*(K166E) expression vector (pPSV38); Gent^R	This study
pLSM166	*P. aeruginosa* P_{lacUV5}-*Pa-mraY*(A225V) expression vector (pPSV38); Gent^R	This study
pLSM167	*P. aeruginosa* P_{lacUV5}-*Pa-mraY*(V347M) expression vector (pPSV38); Gent^R	This study
pLSM168	*P. aeruginosa* P_{lacUV5}-*Pa-mraY*(F224S) expression vector (pPSV38); Gent^R	This study
pLSM169	*P. aeruginosa* P_{lacUV5}-*Pa-mraY*(Q286R) expression vector (pPSV38); Gent^R	This study
pLSM170	*P. aeruginosa* P_{lacUV5}-*Pa-mraY*(K14E) expression vector (pPSV38); Gent^R	This study
pLSM171	*P. aeruginosa* P_{lacUV5}-*Pa-mraY*(L160R) expression vector (pPSV38); Gent^R	This study
pLSM176	*P. aeruginosa* P_{lacUV5}-*Pa-mraY-VSVG* expression vector (pPSV38); Gent^R	This study
pLSM177	*P. aeruginosa* P_{lacUV5}-*Pa-mraY*^{T23P}-*VSVG* expression vector (pPSV38); Gent^R	This study
pLSM196	*P. aeruginosa* P_{lacUV5}-*Pa-mraY-D267A* expression vector (pPSV38); Gent^R	This study
pSLM197	*P. aeruginosa* P_{lacUV5}-*Pa-mraY-T23P D267A* expression vector (pPSV38); Gent^R	This study

*Abbreviations: Carb^R, ampicillin/carbenicillin resistance; Cm^R, chloramphenicol resistance; Gent^R, gentamycin resistance; Kan^R, kanamycin resistance

SI METHODS

Plasmid construction

pNG93 [P_{lacUV5}::*Pa-mraY*(*PA4415*)] is a pPSV38 derivative. pPSV38 was digested with EcoRI/XmaI to generate the plasmid backbone. *P. aeruginosa mraY* (PA4415; M1-R360) was amplified from PAO1 gDNA with oNG338/oNG339 to introduce a synthetic RBS (5' - GAGGAGGATACAT - 3'). After digestion with EcoRI/XmaI, the PCR product was ligated into pPSV38 to generate pNG93. The final construct was sequence verified using primers 556 and 557. The *mraY* gene in this and all related constructs below is inducible with IPTG.

pNG102 [P_{lacUV5}::*Pa-mraY(T23P)*] is a pPSV38 derivative. pPSV38 was digested with EcoRI/XmaI to generate the plasmid backbone. *P. aeruginosa mraY* T23P was amplified from PA760 via colony PCR with oNG338/oNG339 to introduce a synthetic RBS (5' -

bioRxiv preprint doi: https://doi.org/10.1101/2023.08.01.551478; this version posted August 1, 2023. The copyright holder for this preprint (which was not certified by peer review) is the author/funder, who has granted bioRxiv a license to display the preprint in perpetuity. It is made available under aCC-BY 4.0 International license.

GAGGAGGATACAT - 3').). After digestion with EcoRI/XmaI, the PCR product was ligated into pPSV38 to generate pNG93. The final construct was sequence verified using primers 556 and 557.

pLSM116 [P_{T7}::*H-SUMO-FLAG-Pa-mraY*] is a pCOLADuet derivative. The gene encoding full-length *P. aeruginosa mraY* was amplified from pNG93 using the primers oLSM302 and oLSM303. Using pCOLADuet as a template, the backbone was amplified using oLSM301 and oLSM304. The fragments were joined using Gibson assembly and sequence verified using primers 34 and 2325.

pLSM117 [P_{T7}::*H-SUMO-FLAG-Pa-mraY*^{T23P}] is a pCOLADuet derivative. The gene encoding full-length *P. aeruginosa mraY*^{T23P} was amplified from pNG102 using the primers oLSM302 and oLSM303. Using pCOLADuet as a template, the backbone was amplified using oLSM301 and oLSM304. The fragments were joined using Gibson assembly and sequence verified using primers 34 and 2325.

pLSM124 [P_{lacUV5}::*Ec-mraY*] is a pPSV38 derivative. The gene encoding full-length *E. coli mraY* was amplified from MG1655 genomic DNA using primers oLSM312 and oLSM313. Using pNG93 as a template, the backbone was amplified using oLSM311 and oLSM314. The fragments were joined using Gibson assembly. The final construct was sequence verified using primers 556 and 557.

pLSM125 [P_{lacUV5}::*Ec-mraY(T23P)*] is a pPSV38 derivative. Using pLSM124 as a template, T23 was mutated to P using site directed mutagenesis (QuikChange Lightning, Agilent) using the primers oLSM315 and oLSM316. The final construct was sequence verified using primers 556 and 557.

pLSM141 [P_{lac}::*Pa-mraY*] is a pRY47 derivative. The gene encoding full-length *P. aeruginosa mraY* was amplified from pNG93 using primers oLSM372 and oLSM373. Using pRY47 as a template, the backbone was amplified using oLSM374 and oLSM368. The fragments were joined using Gibson assembly. The final construct was sequence verified using primers 556 and 48.

pLSM142 [P_{lac}::*Pa-mraY(T23P)*] is a pRY47 derivative. The gene encoding full-length *P. aeruginosa mraY* (T23P) was amplified from pNG102 using primers oLSM372 and oLSM373. Using pRY47 as a template, the backbone was amplified using oLSM374 and oLSM368. The fragments were ligated using Gibson assembly. The final construct was sequence verified using primers 556 and 48.

pLSM143 [P_{lac}::*Ec-mraY*] is a pRY47 derivative. The gene encoding full-length *E. coli mraY* was amplified from pLSM124 using primers oLSM375 and oLSM376. Using pRY47 as a template, the backbone was amplified using oLSM377 and oLSM368. The fragments were ligated using Gibson assembly. The final construct was sequence verified using primers 556 and 48.

bioRxiv preprint doi: https://doi.org/10.1101/2023.08.01.551478; this version posted August 1, 2023. The copyright holder for this preprint (which was not certified by peer review) is the author/funder, who has granted bioRxiv a license to display the preprint in perpetuity. It is made available under aCC-BY 4.0 International license.

pLSM144 [P_{lac}::*Ec-mraY(T23P)*] is a pRY47 derivative. The gene encoding full-length *E. coli mraY* (T23P) was amplified from pLSM125 using primers oLSM375 and oLSM376. Using pRY47 as a template, the backbone was amplified using oLSM377 and oLSM368. The fragments were ligated using Gibson assembly. The final construct was sequence verified using primers 556 and 48.

pLSM176 [P_{lacUV5}::*Pa-mraY*-GS-VSVG] is a pPSV38 derivative. The gene encoding full-length *P. aeruginosa mraY* was amplified from pNG93 using primers oLSM317 and oLSM405. Using pNG93 as a template, the backbone was amplified using oLSM404 and oLSM318. The fragments were ligated using Gibson assembly. The final construct was sequence verified using primers 556 and 557.

pLSM177 [P_{lacUV5}::*Pa-mraY(T23P)*-GS-VSVG] is a pPSV38 derivative. The gene encoding full-length *P. aeruginosa mraY* (T23P) was amplified from pNG102 using primers oLSM317 and oLSM405. Using pNG102 as a template, the backbone was amplified using oLSM404 and oLSM318. The fragments were ligated using Gibson assembly. The final construct was sequence verified using primers 556 and 557.

pLSM196 [P_{lacUV5}::*Pa-mraY*(*D267A)*] is a pPSV38 derivative. Site directed mutagenesis (QuikChange Lightning, Agilent) of pNG93 was performed to make the D267A change using oLSM460 and oLSM461.

pLSM197 [P_{lacUV5}::*Pa-mraY(T23P/D267A)*] is a pPSV38 derivative. Site directed mutagenesis (QuikChange Lightning, Agilent) of pNG102 was performed to make the D267A change using oLSM460 and oLSM461.

Materials
Unless otherwise indicated, all chemicals and reagents were purchased from Sigma-Aldrich. Restriction enzymes were purchased from New England Biolabs. Oligonucleotide primers were purchased from Integrated DNA Technologies.

Bacterial strains, plasmids, oligonucleotide primers and culture conditions
E. coli strains were grown with shaking at 37 ºC in lysogeny broth (LB, 10 g/L tryptone, 5 g/L NaCl, 5 g/L yeast extract), lysogeny broth with no salt (LBNS; 10 g/L tryptone, 5 g/L yeast extract), Terrific Broth (TB; 12 g/L tryptone, 24 g/L yeast extract, 0.4% v/v glycerol, 0.17 M KH_2PO_4, 0.72 M K_2HPO_4), or on LB or LBNS agar as indicated. MM119 was grown at 30ºC. *P. aeruginosa* strains are all derivatives of PAO1 and were grown with shaking at 37ºC in LB, LBNS, Vogel-Bonner minimal media (VBMM; 3.42 g/L trisodium citrate dihydrate, 2.0 g/L citric acid, 10 g/L K_2HPO_4, 3.5 g/L $NaNH_4PO_4$•$4H_2O$, 1 mM $MgSO_4$, 0.1 mM $CaCl_2$) or on LB, LBNS, or VBMM agar as indicated. The following concentration of antibiotics were used to maintain plasmids: ampicillin (Amp), 50 µg/mL; chloramphenicol (Cam), 25 µg/mL; gentamicin (Gent), 15 µg/mL (*E. coli*); Gent, 30 µg/mL (*P. aeruginosa*). The primers, strains and plasmids used in this study are summarized in **SI appendix, Tables 3-5**.

Electroporation of P. aeruginosa

bioRxiv preprint doi: https://doi.org/10.1101/2023.08.01.551478; this version posted August 1, 2023. The copyright holder for this preprint (which was not certified by peer review) is the author/funder, who has granted bioRxiv a license to display the preprint in perpetuity. It is made available under aCC-BY 4.0 International license.

P. aeruginosa strains were made competent using previously described methods[11]. For electroporation, 100 ng of plasmid DNA was added to 40 µL of competent *P. aeruginosa* cells. Transformation was achieved using standard protocols and transformants were selected for using 30 µg/mL Gent.

Viability assays

Overnight cultures of PAO1, PA686, or PA760 derivatives, containing vectors producing the indicated alleles of *mraY* expressed from an IPTG-inducible (P_{lacUV5}) plasmid were normalized to an OD_{600} of 2.4 before being serially diluted. Aliquots (5 µL) of the dilutions were spotted onto LB Gent agar, VBMM Gent agar, with or without IPTG. Plates were incubated at 30°C for 24 h at which point the plates were imaged. A similar protocol was adapted for MG1655 and MM119 derivatives containing vectors producing the indicated alleles of *mraY* from an IPTG inducible (P_{lac}) plasmid.

Immunoblotting

For analysis of protein levels from strains producing MraY-VSVG variants, an overnight culture of each of the strains was allowed to grow in LB containing 30 µg/mL Gent at 37°C. The following day, the cultures were diluted to an OD_{600} of 0.01 and allowed to grow at 37°C in LB containing 30 µg/ml Gent. After 2 h, 1 mM IPTG was added and the cultures were allowed to grow for another 2.5 h. Cultures were normalized to an OD_{600} = 1.0 and cells were collected by centrifugation at 5,000 × g for 2 min. The cell pellet was resuspended in 200 µL of 2× Laemmli buffer, then centrifuged for 10 min at 21,000 x g. Samples were analyzed by SDS-PAGE followed by imunoblotting. Protein was transferred from the SDS-PAGE gel to a nitrocellulose membrane using wet transfer (30 min at 100V) in cold transfer buffer (192 mM glycine, 20% methanol, 25 mM Tris base). The membrane was blocked in 5% (w/v) skim milk powder in Tris-Buffered saline (10 mM Tris-HCl pH 7.5, 150 mM NaCl) containing 0.5% (v/v) Tween-20 (TBS-T) for 45 min at room temperature with gentle agitation. The α-VSVG antibody (V4888, Sigma) was added to the blocking buffer at a 1:5000 dilution for 1 h. The membrane was washed three times in TBS-T for 5 min each before incubation for 1 h with secondary antibody (anti-rabbit IgG HRP, 1:5000 dilution, 7074S, NEB) in TBS-T with 1% (w/v) skim milk powder. The membrane was then washed three times with TBS-T for 5 min each before developing using Clarity Max^{TM} Western ECL Substrate (1705062; BioRad) and imaged using a BioRad ChemiDoc XRS+.

Error Prone PCR

Mutagenesis was adapted from Yang et al[12]. Four independent mutant plasmid libraries were constructed by mutagenizing *mraY* in plasmid pNG93 (P_{lacUV5}::*mraY*) using Taq polymerase with Thermopol buffer (New England Biolabs, M0267L). The forward 5’-ACACTTTATGCTTCCGGCTC-3’ and reverse 5’- ACTGTTGGGAAGGGCGATCAAA-3’ primers were used to amplify *mraY* from pNG93. The resulting PCR products were purified using the Monarch® PCR & DNA Cleanup Kit (NEB, T1030) and used as “megaprimers” that are denatured and annealed to the original plasmid (pNG93) to amplify the vector backbone using Q5® High-Fidelity 2X Master Mix (NEB, M0492S). The reactions were then digested with DpnI to eliminate any remaining parental plasmid DNA. All four libraries were independently electroporated into NEB 10-beta electrocompetent cells (NEB, C3020K) and plated on LB agar supplemented with 15 µg/ml gentamicin at 37ºC overnight.

bioRxiv preprint doi: https://doi.org/10.1101/2023.08.01.551478; this version posted August 1, 2023. The copyright holder for this preprint (which was not certified by peer review) is the author/funder, who has granted bioRxiv a license to display the preprint in perpetuity. It is made available under aCC-BY 4.0 International license.

Transformants were slurried in LB, and the resuspended cells were normalized to an OD_{600} = 10. Cells from 1 mL of resuspension were centrifuged and plasmid DNA was isolated from the cell suspension using the Monarch® Plasmid DNA Miniprep Kit (T1010). All four libraries were independently transformed into electrocompetent PA686 cells and plated on LBNS agar supplemented with 30 µg/ml Gent and grown overnight at 37ºC. The resulting transformant colonies from each of the libraries were slurried in LBNS supplemented with 30 µg/ml Gent. Samples of each were normalized to OD_{60}0 = 10 in LBNS + 10% (v/v) DMSO, and stored at -80ºC. A sample from each library was then thawed and serial dilutions were plated on VBMM 30 µg/ml Gent with or without IPTG [50 µM], and grown at 30ºC overnight. Individual colonies arising on the IPTG supplemented plates from each library were selected and re-streaked on VBMM with or without IPTG. Those that displayed IPTG dependence were further isolated, and the plasmids sent for sequencing. Clones identified to contain a single point mutation were further characterized. The mutated *mraY* genes were each amplified using Q5 High-fidelity polymerase (NEB) via colony PCR. The purified PCR product was digested with EcoRI and XmaI, and subsequently ligated into pPSV38 for validation of the suppression phenotype. All clones were sequence verified. MraY variants are listed in **Table S1**.

Lipid II extraction

Cultures of PAO1, PA686 and MG1655 were grown at 37°C overnight, and MM119 at 30°C overnight. The next day, cultures were diluted to an OD_{600} of 0.01 and allowed to grow for 2 h at the above specified temperatures whereupon 1mM IPTG was added to induce expression of MraY. Cells were collected when the OD_{600} reached ~0.5, and normalized to OD = 1 in a 1 mL volume. Pellets were collected by centrifugation at 21,000 x g and stored at -20°C until needed. Cells were resuspended in 1 mL LB and added to a mixture of 2:1 methanol : chloroform (3.5 mL total) in borosilicate glass tubes (16x100 mm, Fisher Scientific 1495935AA). Samples were vortexed for 1 min to form a single phase. Cell debris was collected by centrifugation for 10 min at 2000 × g, 21°C. The supernatant was transferred to a fresh borosilicate glass tube, and 2 mL of chloroform was added. The supernatant was acidified using 0.1N HCl to pH 1 as determined by pH indicator strips. The samples were vortexed for 1 min and centrifuged for 20 min at 2000 × g at 21°C to form a two-phase system. Using a glass pipette, as much of the aqueous upper layer was removed without disturbing the interface between the aqueous and organic phases and 1 mL methanol was subsequently added to form a single liquid phase upon vortexing. Samples were transferred to 1.5 mL Eppendorf tubes by glass pipette then dried by nitrogen stream at 40°C. Dried samples were dissolved in 150 µL of a mixture of methanol and chloroform (2:1) by vortexing then centrifuged at 21,000×g for 1 minute and dried by nitrogen stream at 40°C. This was repeated with 40 µL organic mixture, and finally crude lipid extracts were dissolved in 10 µL DMSO by vortexing. Extracts were stored at -20°C.

Lipid II hydrolysis

Crude lipid II (LII) extracts were added (5 µL) to 5 µL of 0.2 M HCl, for a final concentration of 0.1 M HCl. Samples were boiled at 100°C for 15 min and then cooled to 4°C in a thermocycler. 10 µL of sodium borate pH 9 was added followed by 1 µL 0.5M NaOH to neutralize the solution. 2 µl of 100 mg/ml sodium borohydride was added and the samples were allowed to incubate for 30 min at room temperature. Following the incubation, 2 µl of 20% phosphoric acid

bioRxiv preprint doi: https://doi.org/10.1101/2023.08.01.551478; this version posted August 1, 2023. The copyright holder for this preprint (which was not certified by peer review) is the author/funder, who has granted bioRxiv a license to display the preprint in perpetuity. It is made available under aCC-BY 4.0 International license.

was added to quench the reaction and the samples were mixed and immediately subjected to LC/MS analysis.

LC/MS

High-resolution LC/MS traces of soluble LII hydrolysis products were obtained using the following protocol. Briefly, the hydrolyzed samples were subjected to LC/MS analysis (ESI, positive mode). A Waters Symmetry Shield RP8 column (3.5 µm, 4.6 mm X 150 mm) was used to separate hydrolysis products using the following gradient (A, H_2O + 0.1% formic acid; B, acetonitrile + 0.1% formic acid; 0.5 ml/min): 0% B for 5 min, followed by a linear gradient of 0%-20% B over 40 min. Data was obtained on an Agilent 6546 LC-q-TOF Mass Spectrometer. Expected ion masses were extracted with a tolerance of 0.01 mass units.

Purification of UDP-MurNAc pentapeptide

Accumulation of the precursor was performed as previously described[13] with the following modifications. *Bacillus cereus* ATCC 14579 was grown in LB-lennox medium at 37°C until the OD_{600} reached between 0.7-0.8, at which point 130 µg/mL of chloramphenicol was added. After 15 minutes of incubation, 5 µg/ml of vancomycin was added and the cells allowed to incubate for another 60 min at 37°C with shaking. The culture was then cooled on ice and harvested by centrifugation (4000 x g, 20 min, SLC-6000 rotor, 4°C). Cells were collected and stored at -20°C until required.

Cells were resuspended in water (0.1 g wet weight/mL) and stirred into boiling water in a flask with stirring. Boiling was allowed to continue for another 15 minutes at which point the flask was removed from heat and allowed to cool to room temperature with stirring. After approximately 20 minutes the resuspension was cooled on ice and the debris was pelleted at 200,000 x g for 60 min at 4°C. The supernatant was removed and lyophilized. The lyophilized material was resuspended in water and acidified to pH 3 using formic acid (1mL/L culture extracted), centrifuged to remove precipitate, and immediately subjected to reversed phase high pressure liquid chromatography (RP-HPLC).

UDP-MurNAc pentapeptide was isolated by RP-HPLC on a Synergi 4u Hydro-RP 80A (250x 10.0 mM). The column was eluted over a 30-min isocratic program (A, H_2O + 0.1 % formic acid; B, acetonitrile + 0.1% formic acid; 4 ml/min), 4% B for 30 min at room temperature. The elution was monitored by UV at 254 nm. UDP-MurNAc-pentapeptide eluted approximately at 20 min in a single peak, which was verified by mass-spectrometry (1194.35 Da). Peak fractions were collected and lyophilized. The final product was resuspended in water for downstream use.

Expression and Purification of PaMraY

For expression of *P. aeruginosa* MraY or MraY^{T23P}, *E. coli* expression strain LSM9 containing pAM174 and the expression plasmid (pLSM116 or pLSM117) was grown in 1L TB supplemented with 2 mM $MgCl_2$, kanamycin, and chloramphenicol at 37°C with shaking until the OD_{600} was 0.7. The cultures were cooled to 20 °C before inducing protein expression with 1mM IPTG and 0.1% (w/v) arabinose. Cells were harvested 19h post induction by centrifugation (6,000 x g, 15 min, 4°C). To purify FLAG-MraY or FLAG-MraY^{T23P}, the cells were resuspended in lysis buffer B (50 mM HEPES pH 7.5, 150 mM NaCl, 20 mM $MgCl_2$, 0.5 mM DTT) and lysed by passage through a cell disruptor (Constant systems) at 25 kpsi twice. Membranes were collected by ultracentrifugation (100,000 x g, 1h, 4°C). The membrane

bioRxiv preprint doi: https://doi.org/10.1101/2023.08.01.551478; this version posted August 1, 2023. The copyright holder for this preprint (which was not certified by peer review) is the author/funder, who has granted bioRxiv a license to display the preprint in perpetuity. It is made available under aCC-BY 4.0 International license.

pellets were resuspended in solubilization buffer B (20 mM HEPES pH 7.0, 0.5M NaCl, 20% (v/v) glycerol, and 1% (w/v) DDM (Thermo Fisher)) and rotated end over end for 1h at 4°C before ultracentrifugation (100,000 x g, 1h, 4°C). The supernatant was supplemented with 2 mM $CaCl_2$ and loaded onto a pre-equilibrated homemade M1 anti-FLAG antibody resin. The resin was washed with 25 column volumes (CVs) of wash buffer C (20 mM HEPES pH 7.0, 0.5M NaCl, 20% (v/v) glycerol, 2 mM $CaCl_2$, 0.1% (w/v) DDM) and the bound protein was eluted from the column with five CVs of elution buffer (20 mM HEPES pH 7.0, 0.5M NaCl, 20% (v/v) glycerol, 0.1% (w/v) DDM, 5 mM EDTA pH 8.0, and 0.2 mg/mL FLAG peptide). Fractions containing the target protein were concentrated and the protein concentration was measured via the Bradford method. Proteins were aliquoted and stored at -80°C until required.

MraY translocase in vitro assay

The assay was performed at 37°C in an assay buffer containing 20 mM HEPES pH 7.5, 500 mM NaCl, 20% (v/v) glycerol, and 0.1% (w/v) DDM, 10 mM $MgCl_2$, 250 µM UDP-MurNAc pentapeptide, and 1.1 mM C55P (Larodan). Protein was added to initiate the reaction at a final concentration of 1.7 µM. At the appropriate time point the reaction was quenched by boiling for 3 min at 95°C. 1.5 units of alkaline phosphatase was added to the sample (NEB M0371L) and incubated at 25°C for 1h. The samples were heat quenched at 65°C to stop the reaction and were immediately loaded for analysis by LCMS. The samples were monitored by UV 254 and by MS (ESI, positive mode). A Thermo Fisher Hypersil Gold aQ C18 (150x4.6 mm 3 µm) HPLC column was used to separate the substrates and products using the following gradient program (A, H_2O + 0.1% formic acid; B, acetonitrile + 0.1% formic acid; 0.4 ml/min): 4% B for 20 min. Data was obtained on an Agilent 6546 LC-q-TOF Mass Spectrometer.

Preparation of lipopolysaccharide and immunoblotting

To isolate LPS from the *P. aeruginosa* strains containing the indicated plasmids, overnight cultures of each of the strains were allowed to grow in LB at 37°C containing 30 µg/mL Gent. The next day, cultures were diluted to an OD_{600} of 0.01 and allowed to grow at 37°C in 25 mL LB containing 30 µg/ml Gent. After 2 h, 1 mM IPTG was added and the cultures were allowed to grow for another 2h until cultures reached mid-log. 20 mL of culture was pelleted at 4000 x g for 12 min, and cells were resuspended in 1 mL LB and the OD_{600} was measured. The cells were pelleted again at 12,000 x g for 2 min, and resuspended in 1X LDS buffer (Invitrogen, NP00008) + 4% BME to an OD_{600} = 20. Samples were boiled at 95 °C for 10 min. Each sample was subjected to the NI Protein Assay (G Biosciences, 786-005) to determine the protein content in each sample. The lysates (50 µl) were then incubated at 55°C with 1.25 µl proteinase K (NEB, P8107S). After 1h of incubation, samples were boiled at 95°C for 10 min, and then frozen at -20°C until required.

Volumes of lysates corresponding to 20 µg of protein were then run on a Criterion XT 4-12% Bis-Tris Precast Gel (Bio-Rad, 3450124) in MES running buffer (50 mM MES, 50 mM Tris base, 1 mM EDTA, 0.1% (w/v) SDS) for 1h 45 min at 100V constant. Glycan was transferred to nitrocellulose membranes as described above with the following differences: membranes were blocked for 1h at room temperature in 1% (w/v) skim milk, and were then incubated with anti-serotype O5 B-band at a 1:1000 dilution overnight at 4 °C (gift from L. Burrows). After three 15-mL TBST washes, membranes were incubated with anti-mouse HRP antibody (1:5000, NEB 7076S) for 1h at room temperature. Blots were developed as described above.

bioRxiv preprint doi: https://doi.org/10.1101/2023.08.01.551478; this version posted August 1, 2023. The copyright holder for this preprint (which was not certified by peer review) is the author/funder, who has granted bioRxiv a license to display the preprint in perpetuity. It is made available under aCC-BY 4.0 International license.

Molecular dynamics simulations

For the coarse-grained MD, the structural model of the *E. coli* MraY dimer was aligned according to the plane of the membrane with memembed[14], and then converted to the Martini 3 force field using the martinize protocol[15]. Bonds of 500 kJ mol^{-1} nm^{-2} were applied between all protein backbone beads within 1 nm. Proteins were built into 13 x 13 nm membranes composed of 40% POPE, and 10% each of POPG, CDL, lipid I, lipid II, C55-P, and C55-PP using the insane protocol[16]. Alternatively, membranes were built with 60% POPE, and 10% each of POPG, CDL, C55-P, and C55-PP. Lipid I, lipid II, C55-P and C55-PP parameters were from Orta et al. [3].Systems were solvated with Martini waters and Na^+ and Cl^- ions to a neutral charge and 0.0375 M. Systems were minimized using the steepest descents method, followed by 1 ns equilibration using 5 fs time steps, then by 100 ns equilibration with 20 fs time steps, before 9 x 10 µs (complex membrane) or 5 x 10 µs (membrane without lipid I or lipid II) production simulations were run using 20 fs time steps, all in the NPT ensemble with the velocity-rescaling thermostat and semi-isotropic Parrinello-Rahman pressure coupling[17,18].

A pose of the *E. coli* MraY dimer with two lipid II molecules bound to the central cavity was selected for further analysis. All non-POPE lipids (except the two bound lipid II molecules) were deleted and the membrane allowed to shrink to 10 x 10 x 10.5 nm over 100 ns with positional restraints applied to the protein backbone. The resulting molecule was then converted to the atomistic CHARMM36m force field[19,20] using the CG2AT2 protocol[21]. Side chain pKas were assessed using propKa3.1[22], and side chain side charge states were set to their default. Production simulations were run for 5 repeats of ca. 510 ns, using a 2 fs time step in the NPT ensemble with the velocity-rescale thermostat and semi-isotropic Parrinello-Rahman pressure coupling[17,18].

All simulations were run in Gromacs 2021.3[23]. Images were made in VMD[24]. Kinetic analysis of protein-lipid interactions and binding site identification were performed using PyLipID[25]. Density and contact analyses of atomistic MD simulations were performed using MDAnalysis[26,27]. Contacts are defined as a distance of less than 4 Angstroms between Lipid II and MraY.

Expression and purification of the YES complex

The YES complex was expressed as described previously[3]. Briefly, Δ*slyD* BL21(DE3) competent cells were transformed with pET22b-$SlyD_{1\text{-}154}$ and pRSFDuet*Ec*MraY-E_{ID21} and plated in LB-agar containing 35 μg/ml Kanamycin and 100 μg/mL Ampicillin. The culture was grown in 2xYT media at 37°C, 225 r.p.m., and induced at an OD_{600} of 0.9 with 0.4mM IPTG at 18°C overnight. The culture was harvested by centrifugation for 10 minutes at 9,000*xg*, 4°C followed by flash freezing.

The cells were lysed using a M-110L microfluidizer (Microfluidics) in 20mM Tris-HCl pH 7.5, 300 mM NaCl, 10% glycerol, 5mM βME, 0.1mM PMSF, 0.1mM benzamidine. The lysate was cleared by a 20-minute centrifugation at 22,000*xg*. The membrane was isolated by ultracentrifugation at 167,424*xg* and solubilized in 10 mM HEPES pH 7.5, 300 mM NaCl, 5% Glycerol, 5mM βME, 0.1mM PMSF, 0.1mM benzamidine, 10 mM imidazole and 1% dodecyl 4-O-α-D-glucopyranosyl-β-D-glucopyranoside (DDM). The extract was cleared by ultracentrifugation then nutated with 1mL NiNTA resin (Qiagen, Alameda, CA) at 4°C for two hours. The resin was washed with five column volumes of wash buffer (10 mM HEPES pH 7.5, 150 mM NaCl, 5% glycerol, 5mM βME, & 0.03% DDM) with 10mM imidazole and eluted in 20mL of wash buffer containing 200 mM

bioRxiv preprint doi: https://doi.org/10.1101/2023.08.01.551478; this version posted August 1, 2023. The copyright holder for this preprint (which was not certified by peer review) is the author/funder, who has granted bioRxiv a license to display the preprint in perpetuity. It is made available under aCC-BY 4.0 International license.

imidazole. The eluent was further purified by SEC (Superdex 200 5/150 GL, Millipore Sigma) in 10mM HEPES pH 7.5, 75 mM NaCl, 5% glycerol, 5mM βME and 0.03% DDM. Fractions were assessed by SDS-PAGE, concentrated, and directly used for cryo-EM sample preparation.

Sample preparation for cryoEM

The protein sample was diluted to a concentration of 5mg/mL in 10mM HEPES pH 7.5, 75 mM NaCl, 2% glycerol, 5mM βME, 0.03% DDM, and 1mM *E. coli* total lipid extract (Avanti Polar Lipids, 100600P). Quantifoil holey carbon films R1.2/1.3 300 Mesh, Copper (Quantifoil, Micro Tools GmbH) grids were glow discharged with a 2-minute 20Å plasma current using a Pelco easiGlow, Emitech K100X. Grids were prepared using a Vitrobot (FEI Vitrobot Mark v4 x2, Mark v3) by applying 3μL of 5mg/mL YES(T23P) complex onto the grid followed by a 3.5 second blot using a +8-blot force and plunge frozen into liquid ethane.

Data acquisition and analysis

Datasets were collected at a 105k magnification with a pixel size of 0.416 Å/pixel using a 300 kV cryo-TEM Krios microscope equipped with a Gatan K3 6k x 4k direct electron detector and a Gatan Energy Filter (slit width 20eV) in super-resolution mode using Serial EM. Movies with 40 frames were recorded with a total exposure dose of 60 e^-/Å^2 and a defocus range of -1.0 to -2.5 μm. A total of 7,083 movies were gain reference and motion corrected using the patch motion correction built in function in cryosparc (v3.3.2) with a two-fold bin that resulted in a pixel size of 0.832 Å/pixel[28]. The contrast transfer function (CTF) was estimated using CTFFIND4[29]. A total of 3,885,223 particles were obtained by template picker using PDBID:8G01 as reference [3]. Four ab-initio models were obtained using 500,000 particles, from which the best and worst volume were used to sort 4x binned particles through heterogeneous refinement.

Iterative rounds of heterogeneous and non-uniform refinement were performed before re-extracting particles using a 2x bin. This process was continued, and the resulting particles were re-extracted using a 1.3x bin. After several rounds of heterogeneous and non-uniform refinement, 575,243 particles were extracted without binning and used to create a map through non-uniform refinement. Using the MraY model from PDBID: 8G01, a mask covering only the density encompassing MraY was created using ChimeraX[30]. Density outside of this mask was removed using particle subtraction, followed by ab-initio modeling. The best fitting map was then used for further refinement using global CTF, heterogeneous, and non-uniform refinement. The final map with a 3.8Å resolution was composed by 287,765 particles and sharpened using the autosharpen module in PHENIX-1.19.2. The data collection, refinement and validation statistics can be found in Table S2.

References

1. Studier, F. W. & Moffatt, B. A. Use of bacteriophage T7 RNA polymerase to direct selective high-level expression of cloned genes. *J Mol Biol* **189**, 113–130 (1986).

2. Meeske, A. J. *et al.* SEDS proteins are a widespread family of bacterial cell wall polymerases. *Nature* **537**, 634–638 (2016).

bioRxiv preprint doi: https://doi.org/10.1101/2023.08.01.551478; this version posted August 1, 2023. The copyright holder for this preprint (which was not certified by peer review) is the author/funder, who has granted bioRxiv a license to display the preprint in perpetuity. It is made available under aCC-BY 4.0 International license.

3. Orta, A. K. *et al.* The mechanism of the phage-encoded protein antibiotic from ΦX174. *Science* **381**, (2023).

4. Guyer, M. S., Reed, R. R., Steitz, J. A. & Low, K. B. Identification of a Sex-factor-affinity Site in E. coli as gamma delta. *Cold Spring Harb Symp Quant Biol* **45**, Pt 1:135-140 (1981).

5. Markovski, M. *et al.* Cofactor bypass variants reveal a conformational control mechanism governing cell wall polymerase activity. *Proc National Acad Sci* **113**, 4788–4793 (2016).

6. Greene, N. G., Fumeaux, C. & Bernhardt, T. G. Conserved mechanism of cell-wall synthase regulation revealed by the identification of a new PBP activator in Pseudomonas aeruginosa. *Proc National Acad Sci* **115**, 3150–3155 (2018).

7. Taguchi, A. *et al.* FtsW is a peptidoglycan polymerase that is functional only in complex with its cognate penicillin-binding protein. *Nat Microbiol* **4**, 587–594 (2019).

8. Qiao, Y. *et al.* Detection of lipid-linked peptidoglycan precursors by exploiting an unexpected transpeptidase reaction. *J Am Chem Soc* **136**, 14678–81 (2014).

9. Vvedenskaya, I. O. *et al.* Growth phase-dependent control of transcription start site selection and gene expression by nanoRNAs. *Gene Dev* **26**, 1498–507 (2012).

10. Yunck, R., Cho, H. & Bernhardt, T. G. Identification of MltG as a potential terminase for peptidoglycan polymerization in bacteria: MltG lytic transglycosylase. *Mol Microbiol* **99**, 700–718 (2015).

11. Choi, K.-H., Kumar, A. & Schweizer, H. P. A 10-min method for preparation of highly electrocompetent Pseudomonas aeruginosa cells: Application for DNA fragment transfer between chromosomes and plasmid transformation. *J Microbiol Meth* **64**, 391–397 (2006).

12. Yang, D. C., Tan, K., Joachimiak, A. & Bernhardt, T. G. A conformational switch controls cell wall-remodelling enzymes required for bacterial cell division. *Mol Microbiol* **85**, 768–781 (2012).

13. Kohlrausch, U. & Höltje, J. One-step purification procedure for UDP-N-acetylmuramyl-peptide murein precursors from Bacillus cereus. *Fems Microbiol Lett* **78**, 253–257 (1991).

14. Nugent, T. & Jones, D. T. Membrane protein orientation and refinement using a knowledge-based statistical potential. *Bmc Bioinformatics* **14**, 276 (2013).

15. Souza, P. C. T. *et al.* Martini 3: a general purpose force field for coarse-grained molecular dynamics. *Nat Methods* **18**, 382–388 (2021).

16. Wassenaar, T. A., Ingólfsson, H. I., Böckmann, R. A., Tieleman, D. P. & Marrink, S. J. Computational Lipidomics with insane: A Versatile Tool for Generating Custom Membranes for Molecular Simulations. *J Chem Theory Comput* **11**, 2144–2155 (2015).

bioRxiv preprint doi: https://doi.org/10.1101/2023.08.01.551478; this version posted August 1, 2023. The copyright holder for this preprint (which was not certified by peer review) is the author/funder, who has granted bioRxiv a license to display the preprint in perpetuity. It is made available under aCC-BY 4.0 International license.

17. Bussi, G., Donadio, D. & Parrinello, M. Canonical sampling through velocity rescaling. *J Chem Phys* **126**, 014101 (2007).

18. Parrinello, M. & Rahman, A. Polymorphic transitions in single crystals: A new molecular dynamics method. *J Appl Phys* **52**, 7182–7190 (1981).

19. Best, R. B. *et al.* Optimization of the Additive CHARMM All-Atom Protein Force Field Targeting Improved Sampling of the Backbone ϕ, ψ and Side-Chain χ1 and χ2 Dihedral Angles. *J Chem Theory Comput* **8**, 3257–3273 (2012).

20. Huang, J. *et al.* CHARMM36m: an improved force field for folded and intrinsically disordered proteins. *Nat Methods* **14**, 71–73 (2017).

21. Vickery, O. N. & Stansfeld, P. J. CG2AT2: an Enhanced Fragment-Based Approach for Serial Multi-scale Molecular Dynamics Simulations. *J Chem Theory Comput* **17**, 6472–6482 (2021).

22. Søndergaard, C. R., Olsson, M. H. M., Rostkowski, M. & Jensen, J. H. Improved Treatment of Ligands and Coupling Effects in Empirical Calculation and Rationalization of pK a Values. *J Chem Theory Comput* **7**, 2284–2295 (2011).

23. Berendsen, H. J. C., Spoel, D. van der & Drunen, R. van. GROMACS: A message-passing parallel molecular dynamics implementation. *Comput Phys Commun* **91**, 43–56 (1995).

24. Humphrey, W., Dalke, A. & Schulten, K. VMD: Visual molecular dynamics. *J Mol Graphics* **14**, 33–38 (1996).

25. Song, W. *et al.* PyLipID: A Python Package for Analysis of Protein–Lipid Interactions from Molecular Dynamics Simulations. *J Chem Theory Comput* **18**, 1188–1201 (2022).

26. Gowers, R. *et al.* MDAnalysis: A Python Package for the Rapid Analysis of Molecular Dynamics Simulations. *Proc. 15th Python Sci. Conf.* 98–105 (2016) doi:10.25080/majora-629e541a-00e.

27. Michaud-Agrawal, N., Denning, E. J., Woolf, T. B. & Beckstein, O. MDAnalysis: A toolkit for the analysis of molecular dynamics simulations. *J. Comput. Chem.* **32**, 2319–2327 (2011).

28. Punjani, A., Rubinstein, J. L., Fleet, D. J. & Brubaker, M. A. cryoSPARC: algorithms for rapid unsupervised cryo-EM structure determination. *Nat Methods* **14**, 290–296 (2017).

29. Rohou, A. & Grigorieff, N. CTFFIND4: Fast and accurate defocus estimation from electron micrographs. *J Struct Biol* **192**, 216–221 (2015).

30. Pettersen, E. F. *et al.* UCSF ChimeraX: Structure visualization for researchers, educators, and developers. *Protein Sci* **30**, 70–82 (2021).
